# Enhanced Production of Soluble *Pyrococcus furiosus* α-Amylase in *Bacillus subtilis* through Chaperone Co-Expression, Heat Treatment and Fermentation Optimization

**DOI:** 10.4014/jmb.2101.01039

**Published:** 2021-03-23

**Authors:** Kang Zhang, Ruiting Tan, Dongbang Yao, Lingqia Su, Yongmei Xia, Jing Wu

**Affiliations:** 1 State Key Laboratory of Food Science and Technology, Jiangnan University, Wuxi 214122, P.R. China; 2 School of Biotechnology and Key Laboratory of Industrial Biotechnology, Ministry of Education, Jiangnan University, Wuxi 214122, P.R. China; 3 International Joint Laboratory on Food Safety, Jiangnan University, Wuxi 214122, P.R. China

**Keywords:** α-Amylase, *Bacillus subtilis*, chaperone, heat treatment, 3-L fermenter cultivation

## Abstract

*Pyrococcus furiosus* α-amylase can hydrolyze α-1,4 linkages in starch and related carbohydrates under hyperthermophilic condition (~ 100°C), showing great potential in a wide range of industrial applications, while its relatively low productivity from heterologous hosts has limited the industrial applications. *Bacillus subtilis*, a gram-positive bacterium, has been widely used in industrial production for its non-pathogenic and powerful secretory characteristics. This study was conducted to increase production of *P. furiosus* α-amylase in *B. subtilis* through three strategies. Initial experiments showed that co-expression of *P. furiosus* molecular chaperone peptidyl-prolyl *cis*-trans isomerase through genomic integration mode, using a CRISPR/Cas9 system, increased soluble amylase production. Therefore, considering that native *P. furiosus* α-amylase is produced within a hyperthermophilic environment and is highly thermostable, heat treatment of intact culture at 90°C for 15 min was performed, thereby greatly increasing soluble amylase production. After optimization of the culture conditions (nitrogen source, carbon source, metal ion, temperature and pH), experiments in a 3-L fermenter yielded a soluble activity of 3,806.7 U/ml, which was 3.3- and 28.2-fold those of a control without heat treatment (1,155.1 U/ml) and an empty expression vector control (135.1 U/ml), respectively. This represents the highest *P. furiosus* α-amylase production reported to date and should promote innovation in the starch liquefaction process and related industrial productions. Meanwhile, heat treatment, which may promote folding of aggregated *P. furiosus* α-amylase into a soluble, active form through the transfer of kinetic energy, may be of general benefit when producing proteins from thermophilic archaea.

## Introduction

Alpha-amylase (E.C. 3.2.1.1) is an enzyme that catalyzes the hydrolysis of α-1,4 linkages in starch and related carbohydrates and has been widely applied in starch processing, baking, brewing and the de-sizing of textiles [1-3]. In industrial starch processing, α-amylases are used in the liquefaction step to hydrolyze starch into glucose oligomers. The ideal temperature and pH for this step are 105°C and pH 4.5, respectively. Since 1980, *Bacillus licheniformis* α-amylase has been the main enzyme used in starch liquefaction. This enzyme exhibits optimal activity at 90°C and pH 6.0, which can only partly satisfy the starch liquefaction requirement [4-6]. Hyperthermophilic enzymes exhibit optimal activity at temperatures higher than 90°C. Most of these enzymes come from thermophilic archaea, like *Pyrococcus furiosus*, *Thermococcus kodakarensis* and *Sulfolobus solfataricus*, while the remainder come from thermophilic bacteria [7-9]. *P. furiosus* α-amylase is a hyperthermophilic enzyme that exhibits optimal activity at about 100°C, where it has a half-life of more than 12 h [10, 11]. In addition to its excellent thermostability, *P. furiosus* α-amylase exhibits optimal activity at a pH of about 5.6, with good stability and no less than 80% of optimal activity at pH values from 4.5 to 6.5. These characteristics make *P. furiosus* α-amylase potentially suitable for industrial applications.

*P. furiosus* α-amylase has been known and characterized since 1990, but the relatively low level of heterologous expression has limited its industrial application [2]. When produced in *Escherichia coli*, *P. furiosus* α-amylase is prone to form inclusion bodies. Thus, the highest reported yield has been 11.4 U/ml [12]. *P. furiosus* α-amylase is also prone to form inclusion bodies when produced in *Bacillus* using a signal peptide with low secretion efficiency. When using a signal peptide with high secretion efficiency, *P. furiosus* α-amylase is prone to be degraded by the quality control proteases HtrA/HtrB, or the remaining extracellular proteases, because it folds slowly after translocation across the cytoplasmic membrane [12-14]. When produced in *Bacillus subtilis* and *Bacillus amyloliquefaciens*, the highest reported yields have been 0.7 and 2,714 U/ml, respectively. The highest reported production of *P. furiosus* α-amylase in yeast yielded 220 U/ml [15]. The relatively low production levels of *P. furiosus* α-amylase in these mesophilic host strains may be caused by poor protein folding in these strains.

Thermophilic archaea and mesophilic prokaryotic and eukaryotic microbes all express molecular chaperone systems that assist protein folding, but they differ in type, structure, and number [16, 17]. For instance, they all contain ATP-dependent chaperonins of similar structure (two stacked, back-to-back rings), but the kind and number of subunits in the rings are different [18]. Furthermore, prefoldins are a kind of important molecular chaperones that direct unfolded or misfolded proteins to chaperonins for correct folding. They exist as a hetero-hexameric assembly with a jellyfish-like structure and function in an ATP-independent manner. Prefoldins have been found only in archaea and eukaryotic microbes [19, 20]. A novel prefoldin recently found in *Methanocaldococcus jannaschii*, a thermophilic archaean, is a homohexamer that assembles into a filamentous structure. This protein exhibits activity similar to those of the hetero-hexameric prefoldins [21, 22]. Peptidyl-prolyl *cis-trans* isomerase (PPIase) is another important type of chaperone. These enzymes catalyze the *cis-trans* isomerization of prolyl imide bonds in protein, which is a rate-limiting step in protein folding [23]. PPIases have been found in archaea as well as prokaryotic and eukaryotic microbes [24, 25]. These differences among the chaperone systems of thermophilic archaea, mesophilic prokaryotic and eukaryotic microbes may be among the reasons for the poor folding of thermophilic archaeal enzymes like *P. furiosus* α-amylase in commonly used mesophilic prokaryotic and eukaryotic host strains.

Many strategies have been employed to convert the *P. furiosus* α-amylase inclusion bodies produced in *E. coli* into soluble, active enzyme. They include co-expression of heterologous chaperones, like *P. furiosus* prefoldins, chaperonins and small heat shock proteins, and overexpression of homologous chaperones like GroEL/GroES. Both of these strategies have been shown to reduce the level of inclusion body formation and increase soluble activity, but co-expression of *P. furiosus* prefoldins has given the best results [26]. Other strategies include solubilization of the inclusion bodies found within cells disrupted using ultrasound. This has been accomplished through heat treatment or glycerol extraction of the sediment, followed by purification through Phenyl Sepharose chromatography, which cannot be scaled up for industrial production [27, 28]. Even when the production bottleneck of *E. coli* is improved using these methods, *E. coli* presents a food safety issue that restricts its application in the food processing industry.

**Fig. 1:**
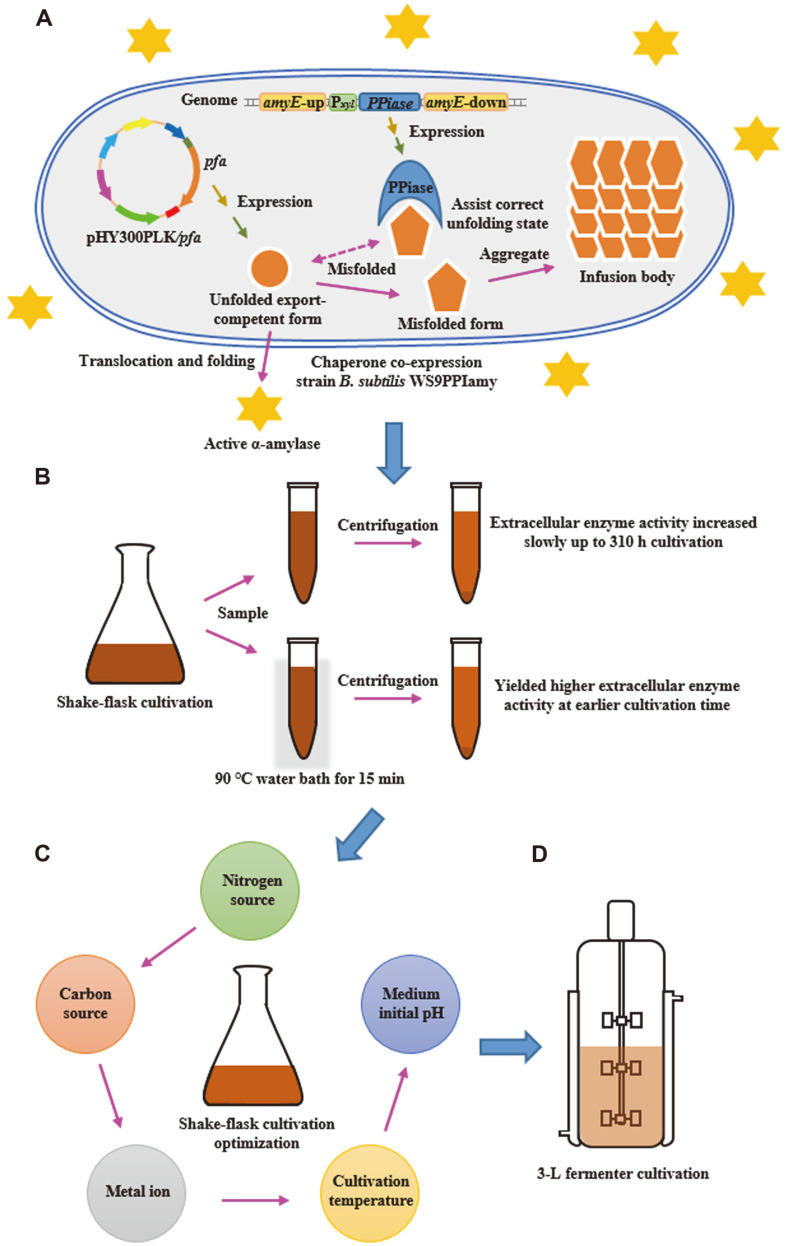
Scheme of strategies used to improve *P. furiosus* α-amylase production in *B. subtilis*. (**A**) Co-expression of molecular chaperone from thermophilic archaea in *B. subtilis*. (**B**) Heat treatment of the CB. (**C**) Shake-flask cultivation optimization. (**D**) 3-L fermenter cultivation.

*B. subtilis* shows great potential for the production of *P. furiosus* α-amylase because it is non-pathogenic, has powerful secretory ability and can be grown to high cell density [29, 30]. In our previous study (unpublished), *P. furiosus* α-amylase production in *B. subtilis* was improved from 18.3 to 135.1 U/mL through signal peptide optimization and overexpression of the extracellular chaperone PrsA. However, even in this system substantial amounts of inclusion bodies could be found in the cytoplasm. In this study, *P. furiosus* α-amylase production in *B. subtilis* was improved using three strategies (Fig. 1). Firstly, co-expression of molecular chaperones from thermophilic archaea was assessed. Then, heat treatment of the culture broth (CB) was investigated. The heat-treated samples exhibited higher soluble activity and their own extraordinary stability, like that of untreated extracellular enzyme, during storage. Finally, shake-flask cultivation conditions were optimized and production was scaled up to a 3-L fermenter.

## Materials and Methods

### Materials

Yeast extract powder 1, industrial yeast extract powder 2 and peptone 1 were purchased from Angie Yeast Co., Ltd. (China). Soy meal 1 was purchased from Shandong Longkete Enzyme Preparations Co., Ltd. (China). Soy peptone and soy meal 2 were purchased from Xi Wang Co., Ltd. (China). Yeast extract, peptone, industrial peptone, fish meal peptone, beef extract fermentation, cottonseed meal, bone peptone, tryptone, beef meal, corn steep powder, yeast extract fermentation and beef peptone were purchased from Sinopharm Chemical Reagent Co., Ltd. (China).

### Strains and Plasmids

The strains and plasmids used in this study are listed in Table 1. *E. coli* JM109 was used as the cloning host for routine plasmid construction. Construction of *B. subtilis* WS9, used as an expression host and stored in our library, has been described in the literature [31]. The *P. furiosus* α-amylase expression plasmid pHY300PLK/*pfa*, which contains the dual promoter P_HpaII_-P_amyQ’_, a mutant *B. subtilis* signal peptide *AspB* (Table S1), and a codon-optimized (*B. subtilis* codon usage) α-amylase gene (GenBank No. MT988065), were constructed previously and stored in our laboratory (Fig. S1 and Table S2). *P. furiosus* α-amylase-producing strain *B. subtilis* WS9amy, constructed through transforming plasmid pHY300PLK/*pfa* into *B. subtilis* WS9, was stored in our laboratory. Codon-optimized (*B. subtilis* codon usage) *M. jannaschii* prefoldins gene (GenBank No. MT988066, Table S2) and *P. furiosus* PPIase gene (GenBank No. MT988067, Table S2) were synthesized and inserted into cloning vector pMD18-T. Construction of the CRISPR/Cas9 disruption plasmid pHYcas9dapr has been described in the literature [32].

### Growth Media and Transformation

LB medium containing (g/l) NaCl 10, peptone 10, and yeast extract 5, was used for routine *E. coli* JM109 and *B. subtilis* cultivation. TB medium containing (g/l) KH_2_PO_4_ 2.3, K_2_HPO_4_ 16.4, glycerol 5, yeast extract 24, and peptone 12, was used for *B. subtilis* shake-flask fermentation. The medium used for 3-L fermenter cultivation contained (g/l) soy peptone 18, yeast extract powder 9, glucose 5, (NH_4_)_2_SO_4_ 2.7, (NH_4_)_2_-H-citrate 1, NaH_2_PO_4_•H_2_O 4, K_2_HPO_4_ 14.6, MgSO_4_•7H_2_O 1, Na_2_SO_3_ 2, AlCl_3_ 0.4, and trace element solution 3 ml/l [33]. The trace element solution contained (g/l) CaCl_2_ 0.5, ZnSO_4_•7H_2_O 0.18, MnSO_4_•H_2_O 0.1, Na2-EDTA 10.05, FeCl_3_ 8.35, CuSO_4_•5H_2_O 0.16, and C°Cl_2_•6H_2_O 0.18. The feeding solution used for 3-L fermenter cultivation contained (g/l) glucose 500, soy peptone 66.7, yeast extract powder 33.3, MgSO_4_•7H_2_O 7.9, and trace element solution 40 ml/l. The media described were supplemented with 100 mg/l ampicillin or 20 mg/l tetracycline as needed. Expression plasmid and CRISPR/Cas9 gene insertion plasmid were transformed into *B. subtilis* using the chemical method of Anagnostopoulos and Spizizen, which is based on the mechanism whereby *B. subtilis* cell membrane and cell wall form defects under the effects of transformation cultivation medium and condition [34].

### Cultivation Conditions

**Shake-flask cultivations.** For routine plasmid construction and seed culture, *E. coli* JM109 or *B. subtilis* strains in 10 ml or 50 ml of LB medium were inoculated with a 0.2% (v/v) portion of inoculum, and then cultivated at 37°C and 200 rpm for 10 h. For shake-flask fermentation, 50 ml of TB medium was inoculated with a 5% (v/v) portion of the seed culture described above and cultivated at 37°C and 200 rpm for 2 h, and then 33°C and 200 rpm for 60 h.

**3-L fermenter cultivations.** A 3-L fermenter (Labfors, Infors-HT Co., Switzerland) containing 0.9 L of fermentation medium was inoculated with a 10% (v/v) portion of seed culture. After inoculation, the dissolved oxygen (DO) decreased slowly from 100%, and when it fell below 30%, the impeller speed was automatically adjusted between 300 and 700 rpm, and pure oxygen was automatically injected into the fermenter as needed to maintain the DO at about 30%. Approximately about 7 h after inoculation, the DO exhibited a sudden increase, indicating that the glucose in the medium had been nearly exhausted and feeding solution should be added. The feeding rate was increased slowly from 2 to 9 ml/h, such that the glucose concentration remained below 0.5 g/l. The glucose concentration was measured with a SBA-40C biosensor (Biology Institute of Shandong Academy of Sciences, Jinan, China). The temperature was kept at 33°C, and the pH was maintained at 7.0 through the addition of NH_4_OH and 20% (v/v) H_3_PO_4_. Tetracycline (20 mg/l) was added every 48 h and antifoam was added as needed. Culture samples were collected at certain times.

### Construction of CRISPR/Cas9 Gene Insertion Plasmids and Chaperone Co-Expression Strains

The primers used in this study are listed in Table 2. Plasmid pBE-S194/*prsA2* was constructed through replacing the original promoter of plasmid pBE-S194/*prsA* (P_apr_) with the xylose-inducible promoter P_xyl_ (Fig. S2A). This was accomplished by using primer pair P01/P02 to amplify P_xyl_ and primer pair P03/P04 to amplify the rest of pBE-S194/*prsA*. The fragments were combined by using the OneStep Cloning Kit (Vazyme, China). Plasmid pBE-S194/*prsA2* contains a MluI site upstream and a SacI downstream of the *prsA* gene. Genes encoding *M. jannaschii* prefoldins and *P. furiosus* PPIase were inserted into cloning vector pMD18-T, which contains an MluI site upstream and a SacI site downstream of the inserted sequence. These sites were used to excise the desired genes and replace the *prsA* gene in the plasmid pBE-S194/*prsA2*, yielding plasmids pBE-S194/pre and pBE-S194/PPI, respectively.

CRISPR/Cas9 gene insertion plasmids pHYcas9pre and pHYcas9PPI were constructed by replacing the N20 sequence and homologous repair template of CRISPR/Cas9 disruption plasmid pHYcas9dapr. The N20 sequence of pHYcas9dapr was replaced through inverse PCR with primer pair P05/P06, yielding plasmid pHYcas9damy1. The nucleotide sequences of the promoter P_xyl_, the *prsA* gene and the terminator were inserted between the upstream and downstream segments of *amyE* homologous repair templates through overlap PCR with primer pairs P07/P08, P09/P10 and P11/P12, respectively. The overlap nucleotide sequence was then ligated into the NcoI restriction site of plasmid pET24a, yielding plasmid pET24a/*prsA*. The homologous repair template in plasmid pET24a/*prsA* was amplified using primer pair P13/P14 and was used to replace the original homologous repair template of pHYcas9damy1, yielding plasmid pHYcas9prsA, of which the other part of pHYcas9damy1 was amplified using primer pair P15/P16. Then, the nucleotide sequences of promoter P_xyl_, prefoldins or PPIase encoding gene and terminate were amplified using primer pair P07/P08 and were used to replace the corresponding part of plasmid pHYcas9prsA, yielding plasmids pHYcas9pre and pHYcas9PPI, respectively.

CRISPR/Cas9 gene insertion plasmids pHYcas9pre and pHYcas9PPI were individually transformed into *B. subtilis* WS9 and transformants were spread onto LB agar containing 20 mg/l tetracycline. The gene insertion mutants were identified using colony PCR with primer pair P17/P18. The PCR products of the desired transformants appeared about 2,000 bp longer than those of transformants containing the normal strain in a gel electrophoresis analysis. Promising PCR products were characterized using DNA sequencing. Finally, the mutant strains were cultivated at 51°C for 10 h to cure the CRISPR/Cas9 gene insertion plasmids, yielding host strains *B. subtilis* WS9pre and *B. subtilis* WS9PPI, respectively.

### Biomass Assay

The dry cell weight (DCW) was determined as follows: Cells in a 5 ml sample of CB were pelleted by centrifugation at 13,800 g and 4°C for 5 min. The pellet was resuspended with 0.9% (w/v) NaCl solution, re-pelleted, and then dried to constant weight at 105°C.

### Bacterial Cell Disruption

Bacterial cell disruption was performed using ultrasound as follows: Cells in the CB were pelleted by centrifugation at 13,800 g and 4°C for 5 min, and then resuspended in equal volume of deionized water. Lysozyme (0.9 mg/ml) was added to the suspension and the mixture was incubated at 37°C for 30 min. The mixture was then disrupted in an ice water bath using an Ultrasonic Homogenizer (JY92-IIDN, Ningbo Xinzhi Biotechnology Co., Ltd. China), at power setting of 20%, and pulse mold of working 2 s and stopping 3 s, for 10 min. The yielded solution was centrifuged at 13,800 g and 4°C for 5 min to produce an ultrasonic disruption supernatant (UDS) and disruption pellets. The disruption pellets were resuspended with equal volume of deionized water to produce an ultrasonic disruption sediment solution (UDSS).

Alternatively, bacterial cells were disrupted using the Bacterial Protein Extraction Kit (Beijing ComWin Biotech Co., Ltd., China) as follows: Cells in the CB were pelleted by centrifugation at 13,800 g and 4°C for 5 min. The pelleted cells were resuspended in equal volume of bacterial protein extraction reagent, which contained 0.1 mg/ml of lysozyme and a nonionic detergent. This suspension was incubated at 37°C and 200 rpm for 2 h to disrupt the cells. The yielded solution was centrifuged at 13,800 g and 4°C for 5 min to produce the Bacterial Protein Extraction Kit disruption supernatant (KDS) and disruption pellets. The disruption pellets were resuspended with equal volume of deionized water to produce Bacterial Protein Extraction Kit disruption sediment solution (KDSS).

### Heat Treatment

A certain volume of the CB was centrifuged at 13,800 g and 4°C for 5 min to produce culture media (CM) and bacterial cell pellets (Fig. S3). The bacterial cell pellets were resuspended with equal volume of deionized water to produce bacterial cell solution (BCS). One milliliter of the CB, CM, BCS, UDS, and KDS were heat treated by placing them in a 90°C water bath for 15 min. The heat-treated CM, UDS, and KDS were shaken well and then assayed for enzyme activity. The heat-treated CB and BCS were centrifuged at 13,800 g and 4°C for 5 min. The supernatant was assayed for enzyme activity, and the sediment was resuspended in 1 ml of deionized water, shaken well and assayed for enzyme activity. One milliliter of the UDSS and KDSS was heat treated by placing it in a 90°C water bath for 15 min. The resulting mixture was shaken well and assayed for enzyme activity.

### Enzyme Activity Assay

The α-amylase activity was measured by determining the concentration of reducing sugars liberated from soluble starch. The reaction was initiated by adding 0.1 ml of appropriately diluted enzyme solution to a pre-heated assay mixture containing 1 ml of 1% (w/v) soluble starch and 0.9 ml of sodium citrate buffer (50 mM, pH 5.0). The assay solution was then incubated at 100°C in a boiling water bath for 10 min. The assay was terminated by adding 3 ml of 3,5-dinitrosalicylic acid solution. The resulting mixture was boiled for 7 min, and then immersed in ice water. Finally, the mixture was diluted with 10 ml of deionized water and the absorbance at 540 nm was measured with a spectrophotometer. One unit of α-amylase activity was defined as the amount of enzyme that produced 1 μmol of reducing sugar per minute from soluble starch under the condition described above [11].

### Protein Analysis

Protein analysis was conducted by using sodium dodecyl sulfate-polyacrylamide gel electrophoresis (SDS-PAGE). Samples were analyzed using a 5% stacking gel and a 12.5% separating gel. CB supernatants (20 μl) or UDSs (20 μl) were mixed with 5 μl of SDS-PAGE buffer (5×) and then placed in a boiling water bath for 5 min. Cell ultrasonic disruption sediments of 1 ml CB were mixed with 20 μl of SDS-PAGE buffer (5×), and then placed in a boiling water bath for 5 min. Then, 8 μl of supernatant mixtures and 1 μl of sediment mixtures were loaded onto the gel for analysis. After electrophoresis, the gels were stained with Coomassie Brilliant Blue R-250 dye. To determine the identity of the protein present in inclusion bodies identified using SDS-PAGE analysis, the corresponding band was excised and analyzed using matrix-assisted laser desorption/ionization-tandem time of flight mass spectrometry (MALDI-TOF/TOF) by Shanghai Haoze Biomedical Technology Co., Ltd. (China).

### Statistical Analysis

All experiments were conducted independently at least three times. The results are presented as the mean ± SD. Statistical analyses were conducted using Student’s *t* test, and differences resulting in values of *p* <0.05 were considered statistically significant.

## Results

### Soluble *P. furiosus* α-Amylase Production Is Increased by Co-Expression with Molecular Chaperones

**Fig. 2:**
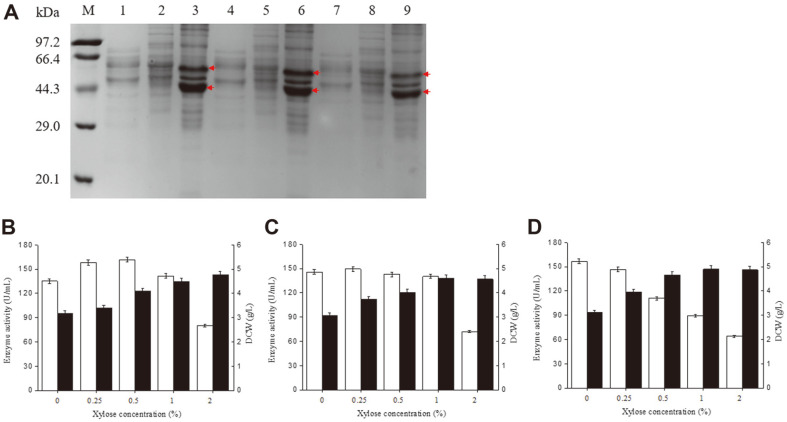
*P. furiosus* α-amylase production in *B. subtilis* strains. (**A**) SDS-PAGE analysis of α-amylase production in *B. subtilis* WS9amy, *B. subtilis* WS9preamy and *B. subtilis* WS9PPIamy. M, protein molecular weight marker; 1, supernatant of CB (*B. subtilis* WS9amy); 2, UDS (*B. subtilis* WS9amy); 3, sediment of cell ultrasonic disruption (*B. subtilis* WS9amy); 4, supernatant of CB (*B. subtilis* WS9preamy); 5, UDS (*B. subtilis* WS9preamy); 6, sediment of cell ultrasonic disruption (*B. subtilis* WS9preamy); 7, supernatant of CB (*B. subtilis* WS9PPIamy); 8, UDS (*B. subtilis* WS9PPIamy); 9, sediment of cell ultrasonic disruption (*B. subtilis* WS9PPIamy). The bands corresponding to α-amylase inclusion bodies are marked with arrows. (**B**-**D**) *P. furiosus* α-amylase production in *B. subtilis* WS9amy (**B**), *B. subtilis* WS9preamy (**C**) and *B. subtilis* WS9PPIamy (**D**) using different xylose concentrations. For all panels: white bar, enzyme activity; black bar, DCW.

In our previous study, substantial amounts of inclusion bodies were formed in the cytoplasm of *B. subtilis* WS9amy, the *P. furiosus* α-amylase-producing strain, which was constructed through transforming expression plasmid pHY300PLK/*pfa* into host strain *B. subtilis* WS9. An SDS-PAGE analysis of these inclusion bodies contained two bands (Fig. 2A). MALDI-TOF/TOF analysis of the material in these bands (Figs. S4 and S5) showed that both contained *P. furiosus* α-amylase, suggesting that *P. furiosus* α-amylase was present as both a monomer and a homodimer in the SDS-PAGE analysis. The relative smaller size of monomer and homodimer than the theoretical size of about 50 and 100 kDa may be related to their partially folded structure under the SDS-PAGE analysis, owing to their excellent thermostability. Similar results have been obtained in previous studies [11, 28].

Initial efforts to reduce inclusion body formation during *P. furiosus* α-amylase production in *B. subtilis* WS9 involved co-expression of *P. furiosus* α-amylase with molecular chaperones from thermophilic archaea (Fig. 1A)[22, 26]. *M. jannaschii* prefoldins and *P. furiosus* PPIase were the molecular chaperones chosen for co-expression studies. Expression of the genes encoding *M. jannaschii* prefoldins and *P. furiosus* PPIase was driven by the B. amyloliquefaciens xylose-inducible promoter P_xyl_. Co-expression cassettes were individually inserted into the *amyE* locus of the *B. subtilis* WS9 genome by using the CRISPR/Cas9 system with gene insertion plasmids pHYcas9pre and pHYcas9PPI (Fig. S2B). This process yielded *B. subtilis* WS9pre, which expresses *M. jannaschii* prefoldins, and *B. subtilis* WS9PPI, which expresses *P. furiosus* PPIase. The *P. furiosus* α-amylase expression plasmid pHY300PLK/*pfa* was transformed into *B. subtilis* WS9pre and *B. subtilis* WS9PPI, yielding *B. subtilis* WS9preamy and *B. subtilis* WS9PPIamy, respectively. The control strain, *B. subtilis* WS9amy, was the parent strain harboring pHY300PLK/*pfa*.

The levels of α-amylase produced by *B. subtilis* WS9amy, *B. subtilis* WS9preamy and *B. subtilis* WS9PPIamy were assessed in shake-flask culture by incubating for 60 h with different concentrations (0%, 0.25%, 0.5%, 1%, and 2%) of xylose. In the absence of added xylose, the extracellular α-amylase activities of *B. subtilis* WS9amy, *B. subtilis* WS9preamy and *B. subtilis* WS9PPIamy were 135.1, 145.5, and 156.7 U/ml, respectively (Figs. 2B-2D). *B. subtilis* WS9amy produced the highest α-amylase activity (161.8 U/ml) with 0.5% xylose, *B. subtilis* WS9preamy produced the highest α-amylase activity (149.4 U/ml) with 0.25% xylose, and *B. subtilis* WS9PPIamy showed the highest α-amylase activity without added xylose. It has been reported that promoter P_xyl_ has weak transcription activity, even in the absence of xylose [35]. Therefore, there must be sufficient basal PPIase expression in *B. subtilis* WS9PPIamy to assist α-amylase in an unfolded export-competent state, and increased PPIase expression had a negative effect on α-amylase production. Since the α-amylase activity produced by *B. subtilis* WS9PPIamy in the absence of added xylose was 1.2-fold greater than that produced by *B. subtilis* WS9amy under the same conditions, and the addition of xylose increases the complexity and cost of fermentation, use of *B. subtilis* WS9PPIamy in the absence of xylose seems the best choice for the industrial production of *P. furiosus* α-amylase.

### Heat Treatment Increases the Production of Soluble, Active *P. furiosus* α-Amylase

**Fig. 3:**
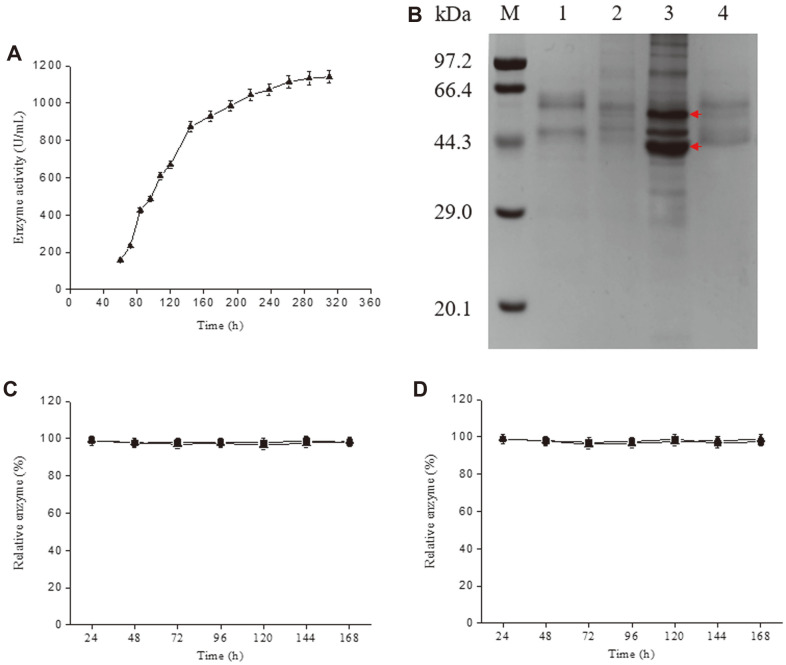
*P. furiosus* α-amylase production and stability. (**A**) α-Amylase production by *B. subtilis* WS9PPIamy over an extended fermentation period. (**B**) SDS-PAGE analysis of α-amylase production in *B. subtilis* WS9PPlamy after shake-flask cultivation for 72 h and heat-treated sample. M, protein molecular weight marker; 1, supernatant of CB; 2, UDS; 3, sediment of cell ultrasonic disruption; 4, supernatant of heat-treated CB. The bands corresponding to α-amylase inclusion bodies are marked with arrows. (**C**) The stability of untreated extracellular α-amylase at 4°C (■) and 25°C (▲). (**D**) The stability of heattreated α-amylase at 4°C (■) and 25°C (▲).

During shake-flask cultivations of *B. subtilis* WS9PPIamy, the extracellular activity increased with fermentation time, reaching 1,141.1 U/ml at 310 h (Fig. 3A). Considering that native *P. furiosus* α-amylase is produced within a hyperthermophilic environment (~100°C) and possesses a high level of molecular rigidity, and that its denaturation requires considerable energy (316 kJ/mol), the correct folding of *P. furiosus* α-amylase may need a high level of energy. Heat treatment can provide energy in a physical way. Meanwhile, the high thermostability of *P. furiosus* α-amylase protects it from thermal deactivation. It seemed reasonable to investigate whether heat treatment can increase the yield of soluble, active *P. furiosus* α-amylase through the transfer of kinetic energy (Fig. 1B).

To test this hypothesis, shake-flask cultivations that had been incubated at 33°C for 60, 72, 96, and 120 h were incubated at 90°C for 30 min, and then the CB supernatants were assayed for α-amylase activity. As shown in Table 3, the enzyme activity increased 2.6- to 7.2-fold after heat treatment, yielding a highest extracellular activity of 1,710.8 U/ml (120 h sample). Indeed, the extracellular activity in the original samples increased substantially from 60 to 120 h, while the activities of the heat-treated samples did not show a correspondingly large increase, especially from 72 to 120 h. The reason for this may be that a substantial portion of the enzyme produced in the first 72 h formed inclusion bodies that slowly unfolded to the export-competent state and was secreted into the CM (Fig. 3B). Heat treatment may accelerate soluble active extracellular production of the hyperthermophilic enzyme by providing kinetic energy.

To determine the optimal heat treatment conditions, shake-flask CBs of *B. subtilis* WS9PPIamy incubated at 33°C for 72 h were incubated at 80°C, 90°C, or 100°C for 0, 5, 15, 30, 60, or 90 min, and then the heat-treated CB supernatants were assayed for α-amylase activity. As show in Table 4, the temperature of the heat treatment did not have a significant effect. Similarly, the enzyme activity present in the CB supernatant did not significantly increase when the duration of the heat treatment exceeded 15 min. Therefore, 90°C for 15 min was selected as the optimal conditions for heat treatment. Under these conditions, the enzyme activity present in the CB supernatant was 6.2-fold greater than that observed in the absence of heat treatment (Fig. 3B).

### Effect of Heat Treatment on α-Amylase Activity in Different Fractions

Shake-flask CBs of WS9PPIamy incubated at 33°C for 72 h were fractionated to determine the amount of α-amylase activity in each fraction and the effect of heat treatment on each (Fig. S3). The results are shown in Table 5. The α-amylase activity of the CB sampled prior to any heat treatment was 393.5 U/ml. Pelleting the cells of CB by centrifugation produced a CM with an α-amylase activity of 233.3 U/ml, and resuspending the cell pellet with equal volume of deionized water yielded a BCS with an α-amylase activity of 149.7 U/ml. Ultrasonic disruption of the BCS, followed by centrifugation, produced a UDS with an α-amylase activity of 124.3 U/ml and a UDSS with an α-amylase activity of 377.5 U/ml. These different fractions were heat treated at 90°C for 15 min separately. When the original CB was sampled after heat treatment, and then centrifuged to produce a heat-treated supernatant and a heat-treated sediment solution, the enzyme activities of the supernatant and sediment solution were 1,456.7 and 438.6 U/ml, respectively. The enzyme activity of heat-treated CM was 292.4 U/ml. When the BCS was heat-treated and centrifuged to produce a heat-treated supernatant and a heat-treated sediment solution, the enzyme activities of the supernatant and sediment solution were 1,102.6 and 204.1 U/ml, respectively. The enzyme activities of heat-treated UDS and UDSS were 87.9 U/ml and 273.9 U/ml, respectively.

For the sake of comparison, the α-amylase activity of heat-treated BCS supernatant (1,102.6 U/ml) was 75.7% of the α-amylase activity of heat-treated CB supernatant (1,456.7 U/ml), while the α-amylase activity of heat-treated CM (292.4 U/ml) was 20.1% of the α-amylase activity of heat-treated CB supernatant (1,456.7 U/ml). These results are consistent with the hypothesis that heat treatment might accelerates the unfolding and export of a part-intracellular *P. furiosus* α-amylase that would otherwise be or remain deposited in inclusion bodies. Furthermore, the sum of α-amylase activities in heat-treated CB supernatant and sediment solution is 1,895.3 U/ml, which is similar with the sum of α-amylase activities in heat-treated CM, heat-treated BCS supernatant, and sediment solution (1,599.1 U/ml), while is much higher than the sum of α-amylase activities in heat-treated CM, UDS and UDSS (654.2 U/ml). These results strongly suggest that whole bacteria must be present for heat treatment to significantly augment α-amylase activity. An alternative explanation, that ultrasonic disruption was too harsh and prevented the heat activation of cellular components, was tested by disrupting the cells using the Bacterial Protein Extraction Kit, which performs a more mild disruption using lysozyme and nonionic detergent. Under these conditions, the α-amylase activities of the KDS and KDSS were 145.6 and 357.8 U/ml, respectively. These results are quite similar to those obtained using ultrasonic disruption. After heat treatment, the enzyme activities of the KDS (148.3 U/ml) was similar with before, while the enzyme activities of the KDSS increased from 357.8 to 515.8 U/ml. Although the total activity of heat-treated CM, KDS and KDS (956.5 U/ml) is still much lower than the activity obtained by heat treating the intact CB, it is 46.2% higher than that of the ultrasonic disruption (654.2 U/ml). These results are partly consistent with the above speculation that ultrasonic disruption was too harsh and prevented the heat activation of cellular components.

### Stability of Heat-Treated Enzyme During Storage

The α-amylase in the supernatant of heat-treated CB was stored at 4°C or 25°C and samples were assayed at regular intervals, during which the untreated extracellular α-amylase was assayed as control. As shown in Figs. 3C, 3D, the heat-treated enzyme remained essentially 100% active over a 168-h period (1 week), similarly with that of untreated enzyme. This extraordinary stability may reflect that the heat-treated enzyme is properly folded just like a normally secreted enzyme.

### Optimization of Shake-Flask Fermentation

**Fig. 4:**
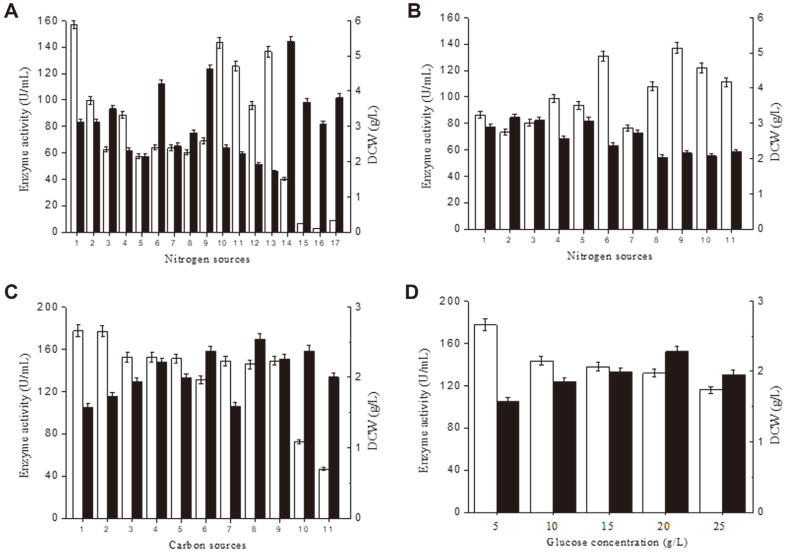
Optimization of the nitrogen and carbon sources in the growth medium. (**A**) The effects of different of nitrogen sources. 1, TB; 2, yeast extract powder; 3, industrial yeast extract powder; 4, yeast extract fermentation; 5, fish meal peptone; 6, soy peptone; 7, bone peptone; 8, peptone 1; 9, industrial peptone; 10, tryptone; 11, beef peptone; 12, beef meal; 13, beef extract fermentation; 14, corn steep powder; 15, cottonseed meal; 16, soy meal 1; 17, soy meal 2. (**B**) Effect of varying the of soy peptone to yeast extract powder concentration (g/l) ratio. 1, 21 and 12; 2, 21 and 9; 3, 21 and 6; 4, 18 and 15; 5, 18 and 12; 6, 18 and 9; 7, 18 and 6; 8, 18 and 3; 9, 15 and 12; 10, 15 and 9; 11, 15 and 6. (**C**) The effects of different of carbon sources. 1, glucose; 2, fructose; 3, maltose; 4, maltodextrin; 5, lactose; 6, glycerol; 7, xylose; 8, sucrose; 9, potato starch; 10, soluble starch; 11, waxy corn starch. (**D**) Effect of varying the glucose concentration. For all panels: white bar, enzyme activity; black bar, DCW.

To further improve *P. furiosus* α-amylase production by *B. subtilis* WS9PPIamy, the culture conditions were optimized in shake-flask experiments with a fixed fermentation time of 60 h, during which the extracellular α-amylase activities were measured (Fig. 1C). In initial experiments, the yeast extract and peptone present in TB medium were replaced with 36 g/l concentrations of seventeen different nitrogen sources including yeast extract powder, industrial yeast extract powder, yeast extract fermentation, fish meal peptone, soy peptone, bone peptone, peptone 1, industrial peptone, tryptone, beef peptone, beef meal, beef extract fermentation, corn steep powder, cottonseed meal, soy meal 1, and soy meal 2. The greatest α-amylase activities were obtained with tryptone (143.1 U/ml), beef extract fermentation (136.6 U/ml), beef peptone (125.4 U/ml), yeast extract powder (99.8 U/ml), and beef meal (95.9 U/ml). The nitrogen sources yielding the greatest cell mass at 60 h were corn steep powder, industrial peptone and soy peptone, which gave DCW values of 5.4, 4.6, and 4.2 g/l, respectively (Fig. 4A). When both price and enzyme activity yield were taken into consideration, a combination of yeast extract and soy peptone was selected for use in subsequent experiments. The concentrations of soy peptone and yeast extract powder were optimized by keeping their total concentration between 21 and 33 g/l. The highest enzyme activity, 136.8 U/ml, was obtained with 15 g/l soy peptone and 12 g/l yeast extract powder. The next highest enzyme activity, 130.8 U/ml, was obtained with 18 g/l soy peptone and 9 g/l yeast extract powder. The cell masses obtained under these conditions were 2.2 and 2.4 g/l, respectively (Fig. 4B). Considering the higher price of yeast extract powder than soy peptone, 18 g/l soy peptone and 9 g/l yeast extract powder were used as the complex nitrogen source in subsequent optimization experiments.

Eleven different carbon sources were evaluated at a concentration of 5 g/l, including glucose, fructose, maltose, maltodextrin, lactose, glycerol, xylose, sucrose, potato starch, soluble starch and waxy corn starch. Use of glucose yielded the highest enzyme activity, 177.3 U/ml, and a DCW of 1.6 g/l (Fig. 4C). After selecting glucose as the optimal carbon source, its concentration was optimized using experiments containing 5, 10, 15, 20, and 25 g/l glucose. The highest α-amylase activity was obtained with a glucose concentration of 5 g/l (Fig. 4D). Thus, the medium employed in subsequent optimization experiments included 5 g/l glucose.

**Fig. 5:**
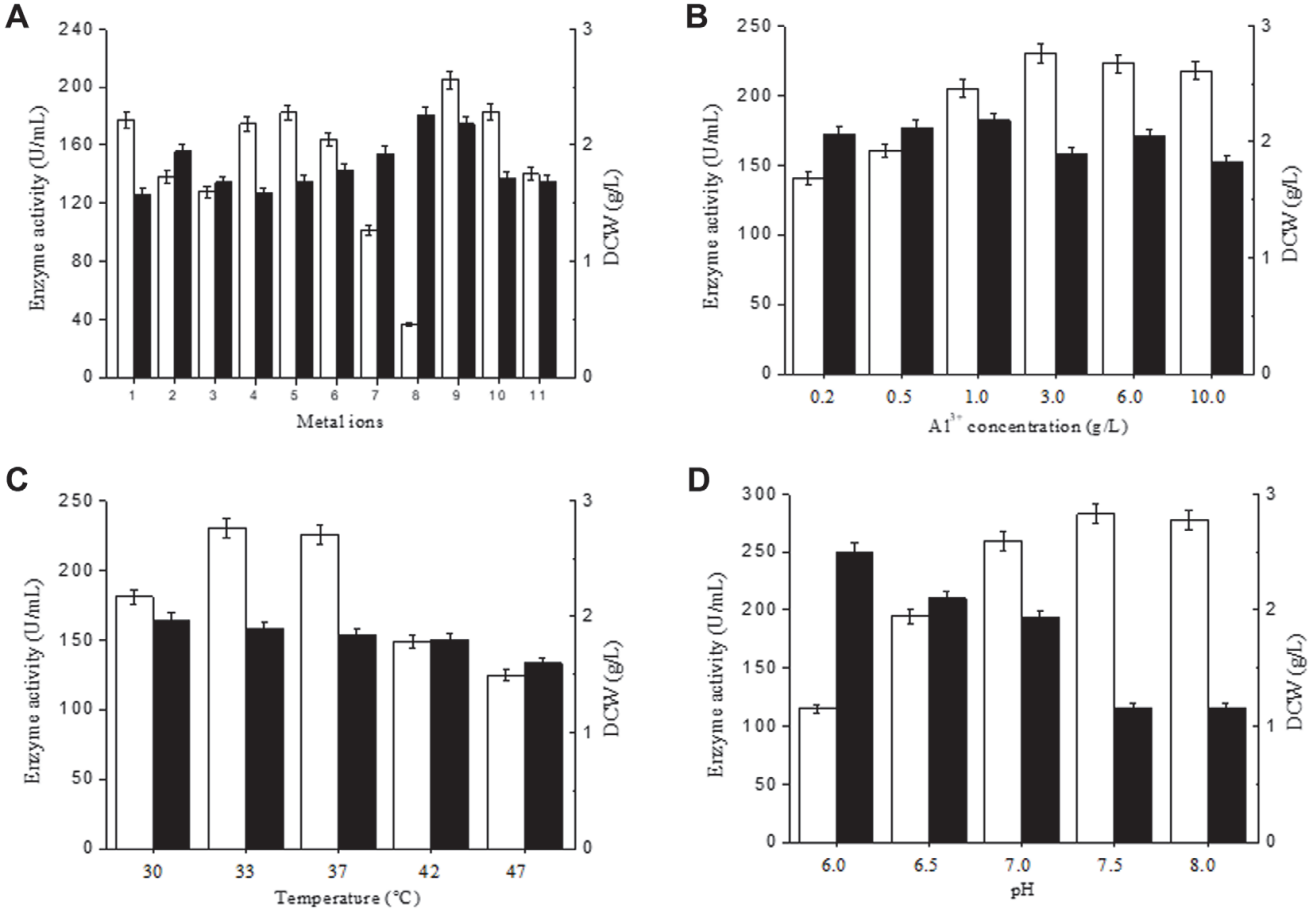
The effects of added metal ions, pH and temperature on *P. furiosus* α-amylase production. (**A**) The effects of different metal ions added at 1 mmol/L concentration. 1, control; 2, Fe^3+^; 3, Fe^2+^; 4, Mn^2+^; 5, Mg^2+^; 6, Ca^2+^; 7, Co^2+^; 8, Cu^2+^; 9, Al^3+^; 10, Ba^2+^; 11, Zn^2+^. (**B**) Effect of varying the Al^3+^ concentration. (**C**) The effect of varying the temperature. (**D**) The effect of varying the pH. For all panels: white bar, enzyme activity; black bar, DCW.

To assess the effect of metal ions on α-amylase production cell growth, 1 mM concentrations of Fe^3+^, Fe^2+^, Mn^2+^, Mg^2+^, Ca^2+^, Co^2+^, Cu^2+^, Al^3+^, Ba^2+^, and Zn^2+^ were added, separately, to the medium. The results are presented in Fig. 5A. The highest α-amylase activity, 204.9 U/ml, was obtained in the presence of Al^3+^. The DCW under these conditions was 2.2 g/l. The optimal Al^3+^ concentration was investigated by adding 0.2, 0.5, 1, 3, 6, and 10 mmol/l Al^3+^. The highest α-amylase activity, 230.0 U/ml, was obtained with 3 mmol/l Al^3+^, which yielded a DCW of 1.9 g/l (Fig. 5B). The medium used in subsequent optimization experiments contained 3 mmol/l Al^3+^.

The fermentation temperature was assessed using cultures at 30, 33, 37, 42, and 47°C. The highest α-amylase activity of 230.0 U/ml was obtained from cultures incubated at 33°C, which yielded a DCW of 1.9 g/l (Fig. 5C). Subsequent experiments were conducted at this temperature. The initial pH of the medium was assessed using media at pH 6.0, 6.5, 7.0, 7.5, and 8.0. The highest α-amylase activity, 282.6 U/ml, was obtained at pH 7.5. At this pH, a DCW of 1.2 g/l was obtained (Fig. 5D). At pH 7.0, the α-amylase activity was 259.4 U/ml (91.8% of that obtained at pH 7.5) and the DCW was 1.9 g/l (158.3% of that obtained at pH 7.5). These results show that use of pH 7.0 would yield better high-density fermentation results than pH 7.5.

Based on these experiments, scale-up fermentation was designed to include a fermentation medium containing soy peptone (18 g/l) and yeast extract powder (9 g/l) as its complex nitrogen source, glucose (5 g/l) as its carbon source, and Al^3+^ (3 mmol/l). Fermentation would be conducted at 33°C and pH 7.0.

### 3-L Fermenter Cultivation

**Fig. 6:**
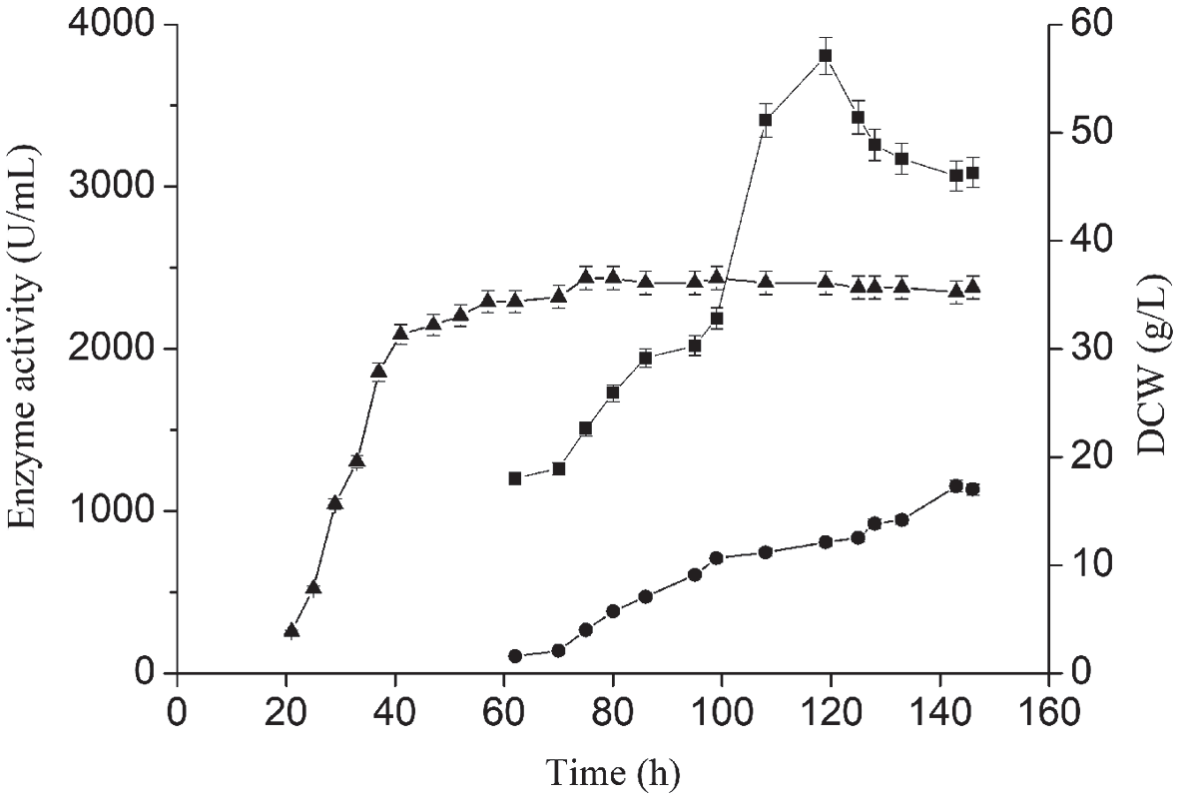
*P. furiosus* α-amylase production in a 3-L fermenter. ●, extracellular α-amylase activity prior to heat treatment; ■, extracellular α-amylase activity after heat treatment; ▲, DCW.

Producing *P. furiosus* α-amylase in a 3-L fermenter was envisioned as an initial fermentation stage (ca. 7 h) followed by a second phase in which the culture was sustained using a feeding solution. Both phases were conducted at pH 7.0 and 33°C. The initial medium was that described in the previous section. The feeding contained soy peptone and yeast extract powder in a 2:1 (w/w) ratio with a total concentration of 100 g/l. The feeding solution also contained 500 g/l glucose. During the cultivation process, the glucose concentration was measured through a biosensor and controlled below 0.5 g/l. Beginning at 60 h of culture, samples were assayed for α-amylase before and after heat treatment. The results are presented in Fig. 6. The cells grew rapidly during the first 45 h and reached their highest density (DCW = 36.6 g/l) at 75 h. Extracellular α-amylase activity before heat treatment increased slowly with time and peaked (1,155.1 U/ml) at 143 h. The extracellular α-amylase activity in heat-treated samples rose more sharply and peaked (3,806.7 U/ml) at 119 h.

In 3-L fermenter cultivation, the appearance of peak heat-treated activity before that of untreated activity was similar to the results seen with shake-flask cultures, supporting our model that *P. furiosus* α-amylase export from the cell in an unfolded export-competent state is a slow process that normally leads to inclusion body formation. However, heat treatment adds kinetic energy that might accelerates the process in both shake-flask and 3-L fermenter, causing a part of the α-amylase that would have been lost to inclusion body formation or proteolysis to be exported into the CM as active, properly folded enzyme.

## Discussion

*B. subtilis* has its own powerful secretory ability for its single membrane structure. In order to control the quality of secreted recombinant proteins, *B. subtilis* uses its own quality control proteases HtrA/HtrB, which can degrade unfolded and misfolded proteins in the interface of membrane and cell wall [14]. The expression level of proteases HtrA/HtrB will increase greatly when the recombinant proteins are over-secreted and accumulate in unfolded or misfolded forms, through regulation by the CssR/CssS two-component system [36]. Therefore, secretory proteins quickly fold into the correct structure and use a signal peptide with proper secretory efficiency to avoid stimulation of the CssR/CssS system and causing major degradation [37]. In our previous study (unpublished), after optimizing the signal peptide and overexpression of extracellular chaperone PrsA, the extracellular activity of *P. furiosus* α-amylase increased from 18.3 to 135.1 U/ml, and SDS-PAGE analysis showed that substantial amounts of inclusion bodies formed in the cytoplasm, which was similar with that in *E. coli* [28]. The formation of inclusion bodies in the cytoplasm avoids extracytoplasmic degradation, while it may also hinder the general translocation process that requires the synthesized proteins in an unfolded export-competent state before translocation [38].

In this study, three strategies of thermophilic archaeal chaperone co-expression, heat treatment and fermentation optimization were applied to improve the soluble production of *P. furiosus* α-amylase in *B. subtilis* (Fig. 1). The co-expression of *P. furiosus* PPIase increased extracellular *P. furiosus* α-amylase activity 1.2-fold in the absence of added xylose. PPIase catalyzes the *cis-trans* isomerization of prolyl imide bonds in protein, and is a heterologous analogue of *B. subtilis* chaperone PrsA [23]. Our previous study showed that overexpression of PrsA improved *P. furiosus* α-amylase activity 1.13-fold, which is relatively consistent with the results in this study. Meanwhile, there are some differences between *B. subtilis* chaperone PrsA and *P. furiosus* PPIase. *B. subtilis* chaperone PrsA is mainly located on the outer surface of the cytoplasmic membrane through N-terminal lipo-modification, and its chaperone activity was shown to be mainly dependent on the NC domains that form a hydrophobic bowl-like crevice, and are partly dependent on the PPIase domain. *P. furiosus* PPIase does not have its own signal peptide and was speculated to be an intracellular protein, which may promote intracellular synthesized *P. furiosus* α-amylase to stay in an unfolded export-competent state. The co-expression of *M. jannaschii* prefoldins increased extracellular *P. furiosus* α-amylase activity 1.1-fold in the absence of added xylose, which was relatively less than that of *P. furiosus* PPIase. In archaea, chaperone prefoldins can capture misfolded proteins and deliver them to Type II chaperonin for ATP-dependent folding, and the affinity of prefoldins to chaperonin influences the delivery efficiency [19, 39]. The interaction of *M. jannaschii* prefoldins and type I chaperonin (*B. subtilis*) may be relatively low and influence the delivery efficiency, which demonstrates that chaperones may be co-expressed with their synergistic chaperones to sufficiently function.

Besides the above strategy to convert inclusion bodies to soluble form through improving the intracellular folding state, the strategy of heat treatment was applied subsequently. Since *P. furiosus* α-amylase is natively produced within a hyperthermophilic environment (~100°C) and possesses a high level of molecular rigidity, and as its denaturation requires considerable energy (316 kJ/mol), the correct folding of *P. furiosus* α-amylase may need a high level of energy [7, 40, 41]. Moreover, *P. furiosus* α-amylase is prone to aggregate for its high hydrophobic characteristic, and a substantial portion of *P. furiosus* α-amylase has been synthesized in 72 h as an inclusion body that slowly unfolded to the export-competent state and secreted into the extracellular space. The heat treatment of intact CB may promote this process through providing kinetic energy. Heat treatment at 90°C for 15 min was shown to be enough for heat activation, demonstrating that the refolding of intracellular *P. furiosus* α-amylase at high temperature occurred much more quickly, which was consistent with the previous report [40]. Previous study showed that secretory expression of *P. furiosus* β-glucosidase in Saccharomyces cerevisiae was increased 4.4-fold by increasing the cultivation temperature from 30°C to 40°C, and that the increased cultivation temperature promoted the folding and secretion of intracellular β-glucosidase [42]. The hypothesis was that the structure characteristics of hyperthermophilic proteins including more salt bridges in the multimer interfaces than the mesophilic analogues hinder the correct folding at low temperature, yielding accumulation of misfolded bodies [43]. Therefore, it seems that the poor folding of hyperthermophilic proteins in mesophilic host strains may share some common reasons for their similar structure characteristics, of which the heat treatment might have a commonly positive effect.

The ultrasonic disruption of the bacterial cell was too harsh and prevented the heat activation of cellular components, while the Bacterial Protein Extraction Kit disruption was mild and partly maintained the heat activation of cellular components. Compared with heat treatments of CM and BCS separately or CM and disruption components separately, heat treatment of intact CB shows the best activation effect. Meanwhile, the mold of heat treating intact CB was simple for application in industrial production and the yielded extracellular enzyme shows good storage stability at both 4°C and 25°C.

The shake-flask culture conditions were optimized and included nitrogen source, carbon source, metal ion, fermentation temperature and pH. The 3-L fermenter cultivation condition was then chosen on the base of shake-flask optimization. The highest activity obtained using the 3-L fermenter (3,806.7 U/ml after heat treatment) was 3.3-fold higher than that obtained prior to heat treatment (1,155.1 U/ml) and 28.2-fold higher than that obtained using shake-flask cultivation (135.1 U/ml). This was also much higher than yields previously reported in *E. coli* (11.4 U/ml) [12], B. amyloliquefaciens (2,714 U/ml) [13] and yeast (220 U/ml) [15].

In summary, the extracellular production of *P. furiosus* α-amylase in *B. subtilis* was increased 1.2-fold by co-expression of *P. furiosus* PPIase and 6.2-fold through heat treatment at 90 °C for 15 min. After optimization of the culture conditions using shake-flask cultures, scale-up in a 3-L fermenter yielded the highest level of soluble, extracellular *P. furiosus* α-amylase activity yet reported: 3806.7 U/ml. These results demonstrate the effectiveness of thermophilic archaeal chaperone co-expression and heat treatment on hyperthermophilic protein production by *B. subtilis*. These simple methods may impact the starch processing industry and improve the production of other industrially useful enzymes.

## Supplemental Materials



Supplementary data for this paper are available on-line only at http://jmb.or.kr.

## Figures and Tables

**Table 1:** Strains and plasmids used in this study.

Strain or plasmid	Characteristics	Sources
Strains		
*E. coli* JM109	*supE44, recA1, thi, endA1, gyrA96, hsdR17* Δ (*lac-proAB*)/F’[*lacZ*ΔM15*,* *traD36, lacІ^q^, proAB^+^*]	Takara
*B. subtilis* WS9	Derived from an undomesticated strain, Δ*srfC*, Δ*spoIIAC*, Δ*nprE*, Δ*amyE*, Δ*aprE*, Δ*nprB*, Δ*bpr*, Δ*mpr*, Δ*epr*	[31]
*B. subtilis* WS9pre	*B. subtilis* WS9 derivative, *amyE*::P_*xyl*_-*prefoldins*	This work
*B. subtilis* WS9PPI	*B. subtilis* WS9 derivative, *amyE*::P_*xyl*_-*PPIase*	This work
*B. subtilis* WS9amy	*B. subtilis* WS9 contains recombinant plasmid pHY300PLK/*pfa*	This lab
*B. subtilis* WS9preamy	*B. subtilis* WS9din contains recombinant plasmid pHY300PLK/*pfa*	This work
*B. subtilis* WS9PPIamy	*B. subtilis* WS9PPI contains recombinant plasmid pHY300PLK/*pfa*	This work
Plasmids		
pMD18-T	Amp^r^, cloning vector	Takara
pET24a	Kan^r^, *E. coli* expression vector	Takara
pHY300PLK/*pfa*	Amp^r^ (*E. coli*), Tet^r^ (*B. subtilis* and *E. coli*), *P. furiosus* α-amylase recombinant expression plasmid	This lab
pBE-S194/*prsA*	Amp^r^ (*E. coli*), Tet^r^ (*B. subtilis* and *E. coli*), promoter P_*apr*_, PrsA overexpression plasmid	[44]
pHYcas9dapr	Amp^r^ (*E. coli*), Tet^r^ (*B. subtilis* and *E. coli*), sgRNA and homologous repair template of *aprE* gene, CRISPR/Cas9 disruption plasmid	[32]
pHYcas9pre	Amp^r^ (*E. coli*), Tet^r^ (*B. subtilis* and *E. coli*), sgRNA of *amyE* gene, *M. jannaschii* *prefoldins* gene insertion plasmids	This work
pHYcas9PPI	Amp^r^ (*E. coli*), Tet^r^ (*B. subtilis* and *E. coli*), sgRNA of *amyE* gene, *P. furiosus* *PPIase* gene insertion plasmids	This work

**Table 2:** Primers used in this study.

Primers	Sequence (5’-3’)^a^
P01	ACGTTTTTAAAGGCTTTTAAATCAACGTGATATAGGTTTGCTAACC
P02	GCGATTTTCTTCATACGCGTTGTACATTCACCTCCTTGATTTAAGTG
P03	ACGCGTATGAAGAAAATCGC
P04	TTAAAAGCCTTTAAAAACGTTTTTAAGG
P05	CCATTCTTCATGCATGGAATGTTTTAGAGCTAGAAATAGCAAGTTAA
P06	ATTCCATGCATGAAGAATGGTTATATTTTACATAATCGCGCGC
P07	CGGAACCATTCTTCATGCATATCAACGTGATATAGGTTTGCTAACC
P08	TAACGTATTGAACGACCCCCGGCAGTACCGGCATA
P09	ATAAGAATGCGGCCGCCTGCGTAATAGACTTTCAGGCGT
P10	CTATATCACGTTGATATGCATGAAGAATGGTTCCGC
P11	ATAGTTTAGCGGCCGCCGTACTGCCTGAACGAGAAGC
P12	TATGCCGGTACTGCCGGGGGTCGTTCAATACGTTAAAACACAA
P13	CTTTGCCCAAGCTTCTAGACTGCGTAATAGACTTTCAGGCG
P14	GCTCAATGTTTTCAGCCAGCGTACTGCCTGAACGAGAAGC
P15	CTGGCTGAAAACATTGAGCCTTTGA
P16	TCTAGAAGCTTGGGCAAAGCGTTTTTC
P17	GTAACATGTAAGCCATAAGCCATTCG
P18	GACCGCAGTGATAGCCTGATCTTC

^a^The nucleotide sequences of restriction enzyme sites were underlined.

**Table 3:** Effects of culture time and heat treatment on extracellular α-amylase activity of *B. subtilis* WS9PPIamy.

Time (h)	Enzyme activity (U/mL)	Heat-treated enzyme activity (U/mL)
60	156.7±3.1	1135.0±25.5
72	233.3±6.6	1588.3±30.7
96	484.1±8.9	1690.8±29.6
120	673.2±19.9	1710.8±47.7

**Table 4:** Effects of temperature and heat treatment time on extracellular α-amylase activity (U/ml) of *B. subtilis* WS9PPIamy.

Temp. (℃)	Heat treatment time (min)					

	0	5	15	30	60	90
100	233.3 ±6.6	843.3 ±20.5	1409.0 ±32.9	1468.1 ±27.9	1535.4 ±33.2	1578.9 ±39.1
90	233.3 ±6.6	871.8 ±14.3	1456.7 ±25.4	1588.3 ±30.7	1605.8 ±48.1	1591.3 ±43.2
80	233.3 ±6.6	840.8 ±23.5	1404.8 ±22.9	1443.2 ±34.1	1562.3 ±42.4	1607.9 ±30.1

**Table 5:** Effects of heat treatment on the different fractions of *B. subtilis* WS9PPIamy.

Fractions	Enzyme activity (U/mL)	Heat-treated enzyme activity (U/mL)
CB	393.5±7.3	1456.7±25.4^a^ and 438.6±10.4^b^
CM	233.3±6.6	292.4±4.1
BCS	149.7±4.5	1102.6±13.5^c^ and 204.1±4.8^d^
UDS	124.3±3.0	87.9±2.2
UDSS	377.5±5.4	273.9±4.4
KDS	145.6±3.2	148.3±3.0
KDSS	357.8±6.1	515.8±14.0

^a^The enzyme activity of heat-treated CB supernatant; ^b^the enzyme activity of heat-treated CB sediment solution that was resuspended with equal volume of deionized water; ^c^the enzyme activity of heat-treated BCS supernatant; ^d^the enzyme activity of heat-treated BCS sediment solution that was resuspended with equal volume of deionized water; CB, culture broth; CM, culture medium; BCS, bacterial cell solution; UDS, ultrasonic disruption supernatant; UDSS, ultrasonic disruption sediment solution; KDS, Bacterial Protein Extraction Kit disruption supernatant; KDSS, Bacterial Protein Extraction Kit disruption sediment solution.
