# Supplementary material for: Enhanced Production of Soluble Pyrococcus furiosus α-Amylase in Bacillus subtilis through Chaperone Co-Expression, Heat Treatment and Fermentation Optimization
Source: J Microbiol Biotechnol. 2021 Mar 23;31(4):570–83. doi: 10.4014/jmb.2101.01039 (PMC9723276; doi:10.4014/jmb.2101.01039)
Supplement: Supplementary file 1 [file jmb-31-4-570-supple.pdf]

J. Microbiol. Biotechnol.
https://doi.org/10.4014/jmb.2101.01039

Supplemental table

Table S1 Characteristics of wild-type *B. subtilis* signal peptide *AspB* and its mutant form that used in this study

Signal peptides	Amino acid sequence[a]	Type
Wild-type *AspB*	MKLAKRVSALTPSTTLAITAKA	Sec
Mutant *AspB*	MKLAITAKA	/

[a], the corresponding overlap amino acids in wild-type *B. subtilis* signal peptide *AspB* were underlined.

Table S2 Sequences of plasmid pHY300PLK/*pfa*, codon-optimized (*B. subtilis* codon usage) *M. jannaschii* prefoldins gene and *P. furiosus* PPIase gene that used in this study

Sequence (5'-3')
Plasmid pHY300PLK/*pfa*[a]
AAGCTTCTAGATTCTCAAAAAATACTACCTGTCCCTTGCTGATTTTTAAACGAGCACGAG
AGCAAAACCCCCCTTTGCTGAGGTGGCAGAGGGCAGGTTTTTTTGTTTCTTTTTTCTCGT
AAAAAAAAGAAAGGTCTTAAAGGTTTTATGGTTTTGGTCGGCACTGCCGACAGCCTCGC
AGAGCACACACTTTATGAATATAAAGTATAGTGTGTTATACTTTACTTGGAAGTGGTTGCC
GGAAAGAGCGAAAATGCCTCACATTTGTGCCACCTAAAAAGGAGCGATTTAGGCGGCGT
TCTGTTTCTGCTTCGGTATGTGATTGTGAAGCTGGCTTACAGAAGAGCGGTAAAAGAAGA
AATAAAAAAGAAATCATCTTTTTTGTTTGGAAAGCGAGGGAAGCGTTCACAGTTTCGGG
CAGCTTTTTTTATAGGAACATTGATTTGTATTCACTCTGCCAAGTTGTTTTGATAGAGTGAT
TGTGATAATTTTAAATGTAAGCGTTAACAAAATTCTCCAGTCTTCACATCGGTTTGAAAGG
AGGAAGCGGAAGAATGAAGTAAGAGGGATTTTTGACTCCGAAGTAAGTCTTCAAAAAAT
CAAATAAGGAGTGTCAAGAATGAAACTGGCAATCACAGCGAAAGCGGCGGCCGGTGCA
CATATGGCCAAATACTTAGAACTCGAAGAGGGCGGTGTTATAATGCAAGCATTCTATTGG
GATGTTCCAGGAGGCGGAATCTGGTGGGACCACATCAGAAGCAAGATTCCTGAATGGTA
CGAGGCCGGCATAAGCGCCATCTGGCTGCCACCTCCTTCTAAAGGAATGAGTGGAGGAT
ATAGTATGGGGTACGACCCATACGACTACTTTGACCTGGGTGAATATTATCAGAAAGGCA
CAGTAGAGACACGCTTTGGATCAAAGGAGGAATTAGTTCGGTTGATTCAAACAGCTCAT
GCTTATGGGATTAAGGTTATCGCCGATGTGGTTATCAACCATAGAGCTGGAGGCGATCTTG
AATGGAACCCATTCGTTGGTGATTATACATGGACAGATTTTTCTAAGGTTGCTTCTGGAAA
GTATACTGCGAACTACTTGGACTTCCACCCAAATGAGCTTCACTGTTGTGACGAGGGTAC
CTTTGGTGGGTTCCCGGATATATGTCACCACAAGGAGTGGGATCAATACTGGCTTTGGAA
ATCAAACGAATCTTACGCGGCATATTTACGGAGCATAGGGTTCGACGGGTGGCGTTTCGA
CTACGTGAAGGGATACGGTGCTTGGGTGGTTCGCGACTGGCTGAACTGGTGGGGAGGTT
GGGCAGTCGGAGAGTATTGGGACACGAACGTTGACGCTTTGCTTTCCTGGGCTTACGAG
AGCGGTGCAAAGGTCTTTGACTTTCCACTTTATTATAAGATGGACGAGGCTTTTGATAAC

AATAATATTCCTGCATTAGTATACGCGTTACAAAACGGTCAGACGGTTGTAAGCAGAGATC
CATTCAAGGCAGTTACATTTGTCGCCAACCATGATACGGACATCATTTGGAACAAGTACC
CTGCCTACGCATTTATCTTAACTTACGAGGGTCAACCAGTAATTTTCTATAGAGATTTTGA
AGAATGGTTGAACAAGGATAAACTTATTAACCTCATCTGGATACACGACCACCTGGCTGG
TGGGTCCACGACAATAGTCTACTACGACAACGATGAGTTAATCTTTGTTCGGAACGGAGA
TTCCCGCCGCCCAGGTTTAATCACTTACATAAATCTGTCCCCTAACTGGGTGGGGCGTTG
GGTGTATGTCCCGAAATTTGCAGGTGCCTGTATACATGAGTATACTGGCAATCTTGGAGGC
TGGGTAGATAAACGTGTTGACAGTAGTGGATGGGTTTACTTGGAGGCTCCGCCGCATGAT
CCTGCCAACGGGTACTATGGATATTCCGTGTGGTCATATTGCGGGGTAGGGTAAAAGCTT
GGTAATAAAAAAACACCTCCAAGCTGAGTGCGGGTATCAGCTTGGAGGTGCGTTTATTTT
TTCAGCCGTATGACAAGGTCGGCATCAGGTGTGACAAATACGGTATGCTGGCTGTCATAG
GTGACAAATCCGGGTTTTGCGCCGTTTGGCTTTTTCACATGTCTGATTTTTGTATAATCAA
CAGGCACGGAGCCGGAATCTTTCGCCTTGGAAAAATAAGCGGCGATCGTAGCTGCTTCC
AATATGGATTGTTCATCGGGATCGCTGCTTTTAATCACAACGTGGGAGCCGTCTGTACGTT
CCTAAACTAGTGTTCTTTTCTGTATGAAAATAGTTATTTCGAGTCTCTACGGAAATAGCGA
GAGATGATATACCTAAATAGAGATAAAATCATCTCAAAAAAATGGGTCTACTAAAATATTA
TTCCATCTATTACAATAAATTCACAGAATAGTCTTTTAAGTAAGTCTACTCTGAACTTAAG
CAAAAGGAGAGGGACGCGTATGAAGAAATGGATGATGGCTGCCGCAGTAGTTTCTCTTA
TGGCGTTGTCAGCATGTAGCAATGACGGTTCAGAAGCCATAGTAGAAACGAAGAACGGA
AATATAACGAAAGACGAGTTCTACAACGAGATGAAGGAGAGAGTCGGAAAGAGCGTAC
TTCGGGACCTCATTGACGAGAAAGTTTTGAGTAAGAAATACAAAGTTACAGACGAAGAA
ATCGACCGCGAAATTGAGCGTATAAAGGAAGCGTATGGCACCCAATATGACCTCGCGGTT
CAGCAGAACGGCGAGAAAGTGATACGCGAAATGGTCAAACTCGACTTATTAAGAACTAA
GGCGGCAGTCGAGGATATAAAGGTAACAGAGAAGGAATTAAAAGAGTACTACGATAACT
ACAAGCCTAAAATCCGCGCCTCCCATATTCTCGTAAAAGATGAGAAGACCGCAAAAGAG
GTTAAAGCCAAGTTAGACAAGGGGGAAGATTTCGCTAAATTGGCCAAGGAGTACAGTCA
AGATCCGGGAAGTGCAAGTAATGGAGGTGATCTCGGTTGGTTCGGACCTGGCAAGATGG
TTAAGGAATTTGAGGAAGCGGCCTACAAACTTAAAGTAGGAGAGGTCTCAGACCCAGTG
AAGACGGATTATGGGTATCATATAATTAAAGTCACCGACAAGGAGAAAAAAAAGTCTTTT
AACGAAATGAAAGATGAAATAGCGTTCGAAGTCAAGAGAAATAAATTGGACCCGGCTAC
CATGCAGTCCAAGGTAGACAAACTGGTGAAAGATGCGGGTGTGGAAATCAAAGATAAGG
ACCTTCAGGACGTAATAGGTCAACAGGGTAAACAGTAAGAGCTCGGTACCCTCGAGGGA
TCCGAATTCAAGCTTGTCGACCTGCAGTCTAGACATCACCATCATCACCACTAATGCGGT
AGTTTATCACAGTTAAATTGCTAACGCAGTCAGGCACCGTGTATGAAATCTAACAATGCG
CTCATCGTCATCCTCGGCACCGTCACCCTGGATGCTGTAGGCATAGGCTTGGTTATGCCGG
TACTGCCGGGAATTCCTGTTATAAAAAAAGGATCAATTTTGAACTCTCTCCCAAAGTTGA
TCCCTTAACGATTTAGAAATCCCTTTGAGAATGTTTATATACATTCAAGGTAACCAGCCAA
CTAATGACAATGATTCCTGAAAAAAGTAATAACAAATTACTATACAGATAAGTTGACTGAT
CAACTTCCATAGGTAACAACCTTTGATCAAGTAAGGGTATGGATAATAAACCACCTACAAT
TGCAATACCTGTTCCCTCTGATAAAAAGCTGGTAAAGTTAAGCAAACTCATTCCAGCACC
AGCTTCCTGCTGTTTCAAGCTACTTGAAACAATTGTTGATATAACTGTTTTGGTGAACGA
AAGCCCACCTAAAACAAATACGATTATAATTGTCATGAACCATGATGTTGTTTCTAAAAGA
AAGGAAGCAGTTAAAAAGCTAACAGAAAGAAATGTAACTCCGATGTTTAACACGTATAA
AGGACCTCTTCTATCAACAAGTATCCCACCAATGTAGCCGAAAATAATGACACTCATTGTT
CCAGGGAAAATAATTACACTTCCGATTTCGGCAGTACTTAGCTGGTGAACATCTTTCATCA
TATAAGGAACCATAGAGACAAACCCTGCTACTGTTCCAAATATAATTCCCCCACAAAGAA
CTCCAATCATAAAAGGTATATTTTTCCCTAATCCGGGATCAACAAAAGGATCTGTTACTTT

CCTGATATGTTTTACAAATATCAGGAATGACAGCACGCTAACGATAAGAAAAGAAATGCT
ATATGATGTTGTAAACAACATAAAAAATACAATGCCTACAGACATTAGTATAATTCCTTTG
ATATCAAAATGACCTTTTATCCTTACTTCTTTCTTTAATAATTTCATAAGAAACGGAACAGT
GATAATTGTTATCATAGGAATGAGTAGAAGATAGGACCAATGAATATAATGGGCTATCATT
CCACCAATCGCTGGACCGACTCCTTCTCCCATGGCTACTATCGATCCAATAAGACCAAAT
GCTTTACCCCTATTTTCCTTTGGAATATAGCGCGCAACTACAACCATTACGAGTGCTGGAA
ATGCAGCTGCACCAGCCCCTTGAATAAAACGAGCCATAATAAGTAAGGAAAAGAAAGAA
TGGCCAACAAACCCAATTACCGACCCGAAACAATTTATTATAATTCCAAATAGGAGTAAC
CTTTTGATGCCTAATTGATCAGATAGCTTTCCATATACAGCTGTTCCAATGGAAAAGGTTA
ACATAAAGGCTGTGTTCACCCAGTTTGTACTCGCAGGTGGTTTATTAAAATCATTTGCAAT
ATCAGGTAATGAGACGTTCAAAACCATTTCATTTAATACGCTAAAAAAAGATAAAATGCA
AAGCCAAATTAAAATTTGGTTGTGTCGTAAATTCGATTGTGAATAGGATGTATTCACATTT
CACCCTCCAATAATGAGGGCAGACGTAGTTTATAGGGTTAATGATACGCTTCCCTCTTTTA
ATTGAACCCTGTTACATTCATTATTCATTACACTTCATAATTAATTCCTCCTAAACTTGATTA
AAACATTTTACCACATATAAACTAAGTTTTAAATTCAGTATTTCATCACTTATACAACAATA
TGGCCCGTTTGTTGAACTACTCTTTAATAAAATAATTTTTCCGTTCCCAATTCCACATTGCA
ATAATAGAAAATCCATCTTCATCGGCTTTTTCGTCATCATCTGTATGAATCAAATCGCCTTC
TTCTGTGTCATCAAGGTTTAATTTTTTATGTATTTCTTTTAACAAACCACCATAGGAGATTA
ACCTTTTACGGTGTAAACCTTCCTCCAAATCAGACAAACGTTTCAAATTCTTTTCTTCATC
ATCGGTCATAAAATCCGTATCCTTTACAGGATATTTTGCAGTTTCGTCAATTGCCGATTGTA
TATCCGATTTATATTTATTTTTCGGTCGAATCATTTGAACTTTTACATTTGGATCATAGTCTA
ATTTCATTGCCTTTTTCCAAAATTGAATCCATTGTTTTTGATTCACGTAGTTTTCTGTATTCT
TAAAATAAGTTGGTTCCACACATACCAATACATGCATGTGCTGATTATAAGAATTATCTTTA
TTATTTATTGTCACTTCCGTTGCACGCATAAAACCAACAAGATTTTTATTAATTTTTTTATAT
TGCATCATTCGGCGAAATCCTTGAGCCATATCTGACAAACTCTTATTTAATTCTTCGCCATC
ATAAACATTTTTAACTGTTAATGTGAGAAACAACCAACGAACTGTTGGCTTTTGTTTAATA
ACTTCAGCAACAACCTTTTGTGACTGAATGCCATGTTTCATTGCTCTCCTCCAGTTGCAC
ATTGGACAAAGCCTGGATTTACAAAACCACACTCGATACAACTTTCTTTCGCCTGTTTCA
CGATTTTGTTTATACTCTAATATTTCAGCACAATCTTTTACTCTTTCAGCCTTTTTAAATTCA
AGAATATGCAGAAGTTCAAAGTAATCAACATTAGCGATTTTCTTTTCTCTCCATGGTCTCA
CTTTTCCACTTTTTGTCTTGTCCACTAAAACCCTTGATTTTTCATCTGAATAAATGCTACTA
TTAGGACACATAATATTAAAAGAAACCCCCATCTATTTAGTTATTTGTTTGGTCACTTATAA
CTTTAACAGATGGGGTTTTTCTGTGCAACCAATTTTAAGGGTTTTCCAATACTTTAAAACA
CATACATACCAACACTTCAACGCACCTTTCAGCAACTAAAATAAAAATGACGTTATTTCTA
TATGTATCAAGATAAGAAAGAACAAGTTCAAAACCATCAAAAAAAGACACCTTTTCAGG
TGCTTTTTTTATTTTATAAACTCATTCCCTGATCTCGACTTCGTTCTTTTTTTACCTCTCGGT
TATGAGTTAGTTCAAATTCGTTCTTTTTAGGTTCTAAATCGTGTTTTTCTTGGAATTGTGCT
GTTTTATCCTTTACCTTGTCTACAAACCCCTTAAAAACGTTTTTAAAGGCTTTTAAGCGTC
TGTACGTTCCTTAAGGAATTATTCCTTAGTGCTTTCTAGGTTAATGTCATGATAATAATGGT
TTCTTAGACGTCAGGTGGCACTTTTCGGGGAAATGTCCGCGGAACCCCTATTTGTATTTAT
TTTTCTAAATACATTCAAATATGTATCCGCTCATGAGACAATAACCCTGATAAATGCTTCAA
TAATATTGAAAAAGGAAGAGTATGAGTATTCAACATTTCCGTGTCGCCCTTATTCCCTTTT
TTGCGGCATTTTGCCTTCCTGTTTTTGCTCACCCAGAAACGCTGGTGAAAGTAAAAGATG
CTGAAGATCAGTTGGGTGCACGAGTGGGTTACATCGAACTGGATCTCAACAGCGGTAAG
ATCCTTGAGAGTTTTCGCCCCGAAGAACGTTTTCCAATGATGAGCACTTTTAAAGTTCTG
CTATGTGGCGCGGTATTATCCCGTGTTGACGCCGGGCAAGAGCAACTCGGTCGCCGCATA
CACTATTCTCAGAATGACTTGGTTGAGTACTCACCAGTCACAGAAAAGCATCTTACGGAT

GGCATGACAGTAAGAGAATTATGCAGTGCTGCCATAACCATGAGTGATAACACTGCGGCC
AACTTACTTCTGACAACGATCGGAGGACCGAAGGAGCTAACCGCTTTTTTGCACAACAT
GGGGGATCATGTAACTCGCCTTGATCGTTGGGAACCGGAGCTGAATGAAGCCATACCAA
ACGACGAGCGTGACACCACGATGCCTGCAGCAATGGCAACAACGTTGCGCAAACTATTA
ACTGGCGAACTACTTACTCTAGCTTCCCGGCAACAATTAATAGACTGGATGGAGGCGGAT
AAAGTTGCAGGACCACTTCTGCGCTCGGCCCTTCCGGCTGGCTGGTTTATTGCTGATAAA
TCTGGAGCCGGTGAGCGTGGGTCTCGCGGTATCATTGCAGCACTGGGGCCAGATGGTAA
GCCCTCCCGTATCGTAGTTATCTACACGACGGGGAGTCAGGCAACTATGGATGAACGAAA
TAGACAGATCGCTGAGATAGGTGCCTCACTGATTAAGCATTGGTAACTGTCAGACCAAGT
TTACTCATATATACTTTAGATTGATTTAAAACTTCATTTTTAATTTAAAAGGATCTAGGTGA
AGATCCTTTTTGATAATCTCATGACCAAAATCCCTTAACGTGAGTTTTCGTTCCACTGAGC
GTCAGACCCCTTAATAAGATGATCTTCTTGAGATCGTTTTGGTCTGCGCGTAATCTCTTGC
TCTGAAAACGAAAAAACCGCCTTGCAGGGAGGTTTTTCGAAGGTTCTCTGAGCTACCAA
CTCTTTGAACCGAGGTAACTGGCTTGCAGGAGCGCAGTCACCAAAACTTGTCCTTTCAG
TTTAGCCTTAACCGGCGCATGACTTCAAGACTAACTCCTCTAAATCAATTACCAGTGGCT
GCTGCCAGTGGTGCTTTTGCATGTCTTTCCGGGTTGGACTCAAGACGATAGTTACCGGAT
AAGGCGCAGCGGTCGGACTGAACGGGGGGTTCGTGCATACAGTCCAGCTTGGAGCGAA
CTGCCTACCCGGAACTGAGTGTCAGGCGTGGAATGAGACAAACGCGGCCATAACAGCG
GAATGACACCGGTAAACCGAAAGGCAGGAACAGGAGAGCGCACGAGGGAGCCGCCAG
GGGGAAACGCCTGGTATCTTTATAGTCCTGTCGGGTTTCGCCACCACTGATTTGAGCGTC
AGATTTCGTGATGCTTGTCAGGGGGCGGAGCCTATGGAAAAACGCTTTGCCC

M. jannaschii prefoldins gene

ATGGTCAATGAAGTTATTGACATCAATGAAGCAGTTCGTGCGTATATCGCGCAAATCGAA
GGATTACGGGCGGAAATTGGCCGCCTTGATGCGACGATTGCAACGTTACGCCAATCATTA
GCGACACTTAAATCACTTAAAACATTAGGAGAAGGCAAAACGGTGTTAGTCCCGGTGGG
CTCTATTGCACAAGTCGAAATGAAAGTGGAAAAAATGGATAAAGTGGTTGTGAGCGTCG
GCCAAAATATCTCAGCGGAACTGGAATATGAAGAAGCACTTAAATATATCGAAGATGAAA
TTAAAAAACTGCTGACGTTTAGACTTGTCCTGGAACAAGCGATTGCAGAACTGTATGCG
AAAATCGAGGACCTGATCGCGGAAGCACAACAAACGTCTGAAGAAGAAAAAGCAGAA
GAAGAAGAAAATGAAGAAAAAGCGGAATAA

P. furiosus PPIase gene

ATGAAAGTCGAAAAAGGAGATGTCATTCGTCTGCATTATACGGGCAAAGTCAAAGAAAC
AGGCGAAATCTTTGATACGACCTACGAAGATGTTGCGAAAGAAGCACGTATCTATAATCC
TAATGGCATCTATGGACCTGTCCCTATTGCAGTGGGAGCAGGACATGTGTTACCGGGACT
GGATAAACGCCTTATCGGCCTGGAAGTCAAAAAAAAATATGTGATCGAAGTGCCGCCGG
AAGAAGGCTTTGGCTTGCGTGATCCTGGCAAAATTAAAATTATTCCTCTGGGCAAATTTC
GCAAATCAGGCATTATTCCGTATCCGGGCTTAGAAATTGAAGTCGAAACGGAAAATGGCC
GTAAAATGCGGGGTCGTGTGCTGACGGTCTCAGGAGGCCGCGTGAGAGTGGATTTTAAT
CATCCGTTAGCAGGCAAAACGTTGGTGTATGAAGTGGAAGTTGTCGAAAAAATCGAAGA
TCCGATTGAAAAAATCAAAGCGCTGATTGAACTGCGGTTACCGATGATCGATAAAGATAA
AGTCATCATCGAAATTAGCGAAAAAGATGTCAAACTTAATTTTAAAGATGTGGATATTGAT
CCTAAAACACTTATTCTTGGCGAAATTCTGTTAGAATCAGATCTTAAATTTATCGGCTATG
AAAAAGTGGAATTTGAACCGACGATTGAAGAACTGCTTAAACCTAAATCAGCGGAAGAA
CAAGAATCACCTAATGAAGAACAACAAGAAGAATCTGAATCTAAAGCGGAAGAATCTTA
A

---

[a], the sequence of codon-optimized (*B. subtilis* codon usage) *P. furiosus* α-amylase gene was underlined.

Supplemental figures

Fig. S1 Scheme of *P. furiosus* α-amylase expression plasmid pHY300PLK/*pfa* construction method. Plasmids pBE-S/*pfa*, pBE-S194/*prsA*, and pHYCGTd4 are *P. furiosus* α-amylase secretion signal peptide screening plasmid, *B. subtilis* chaperone PrsA expression plasmid, and *Bacillus circulans* 251 β-CGTase expression plasmid [1], respectively. P_{HpaII}-$P_{amyQ'}$, dual promoter P_{HpaII}-$P_{amyQ'}$; P_{apr}, *B. subtilis* protease Apr promoter; *pfa*, *P. furiosus* α-amylase gene; *prsA*, *B. subtilis* chaperone PrsA gene; *ori*, replication origin

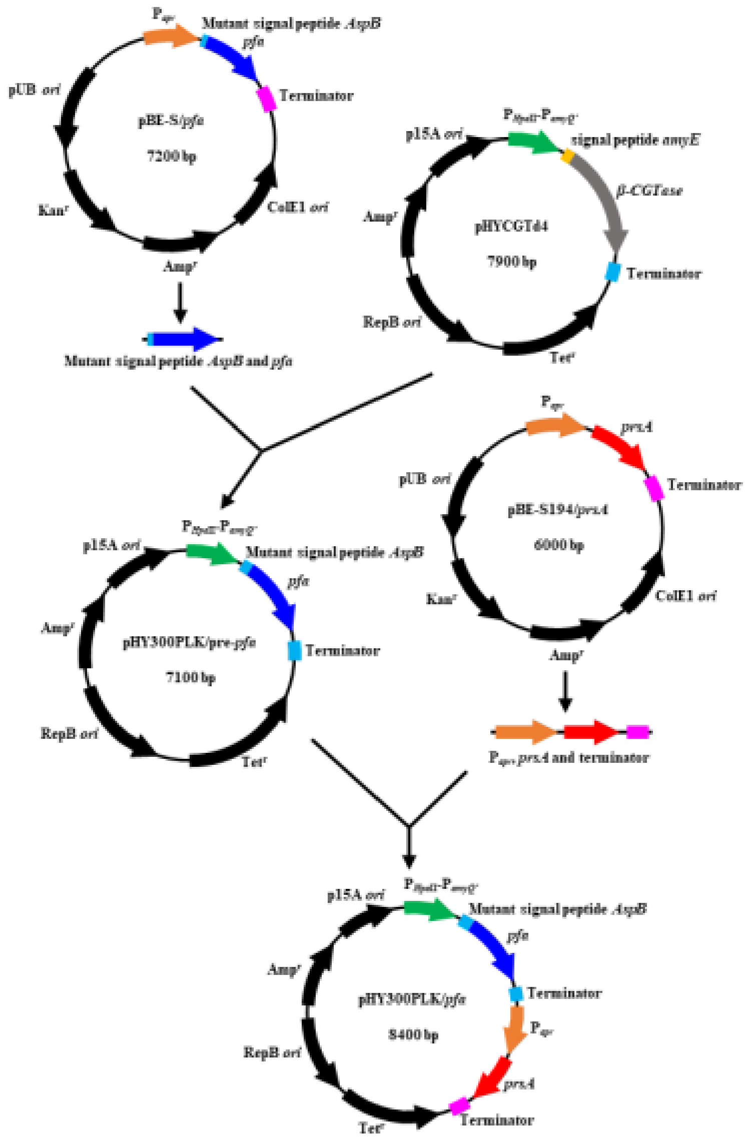

Fig. S2 Schemes of CRISPR/Cas9 gene insertion plasmids construction method (**A**) and genome engineering strategy (**B**). P_{xyl}, *B. amyloliquefaciens* xylose-inducible promoter; *prsA*, *B. subtilis* chaperone PrsA gene; *prefoldins*, *M. jannaschii* chaperone Prefoldins gene; *PPIase*, *P. furiosus* chaperone PPIase gene; *ori*, replication origin; Ter, terminator

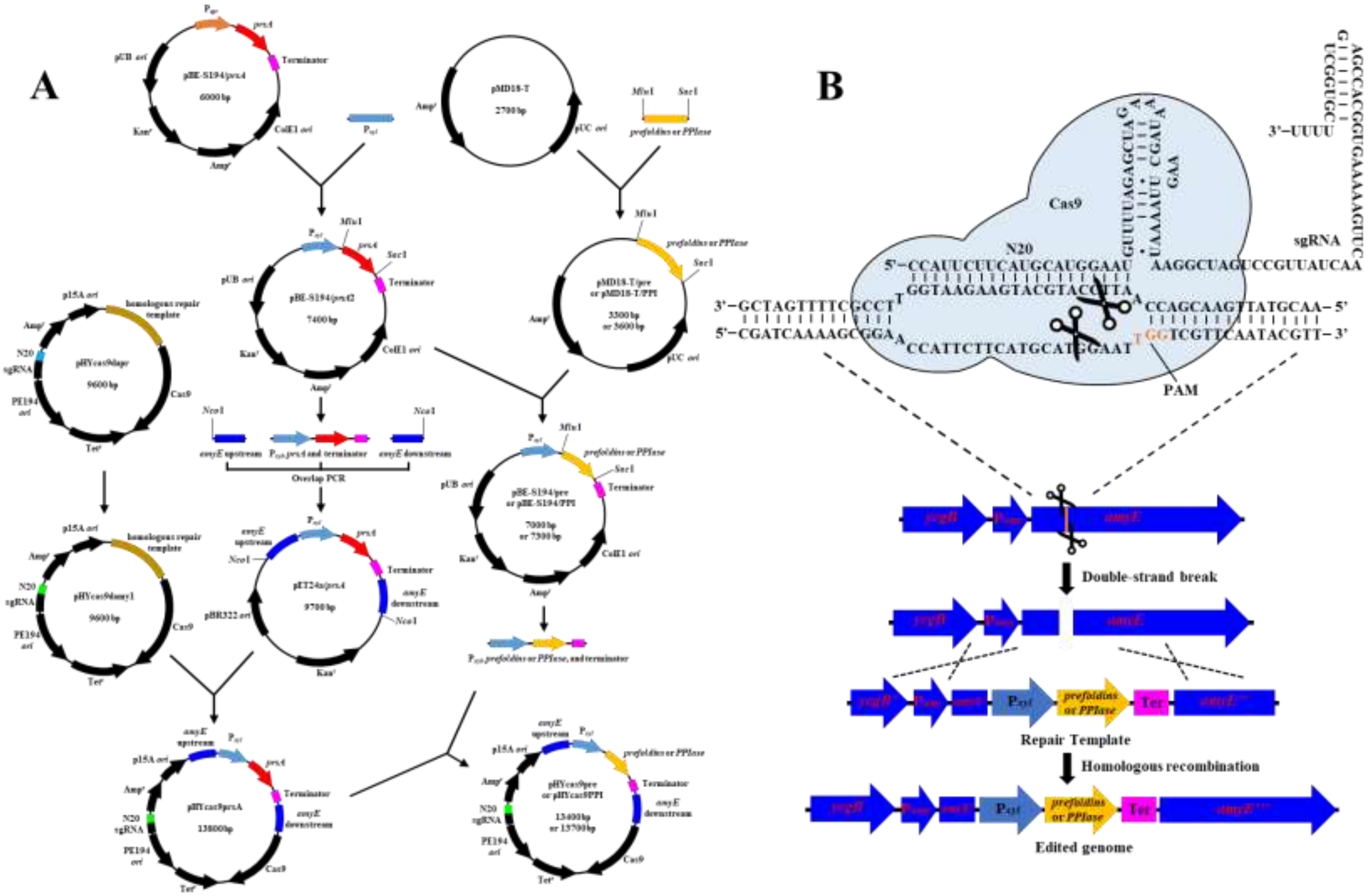
A
B
Cas9
N20
sgRNA
PAM
3'−UUUU
5'−CCAUUCUUCAUGCAUGGAAU
AAGGCUAGUCCGUUAUCAA
GGTAAGAAGTACGTACCTTA
3'−GCTAGTTTTCGCCT
5'−CGATCAAAAGCGGA
CCAGCAAGTTATGCAA−5'
CCATTCTTCATGCATGGAAT
TGGTCGTTCAATACGTT−3'
Double-strand break
Repair Template
Homologous recombination
Edited genome
Overlap PCR
Terminator
Cas9

Fig. S3 Schemes of heat treatment method. CB, culture broth; CM, culture medium; BCS, bacterial cell solution; UDS, ultrasonic disruption supernatant; UDSS, ultrasonic disruption sediment solution; KDS, Bacterial Protein Extraction Kit disruption supernatant; KDSS, Bacterial Protein Extraction Kit disruption sediment solution

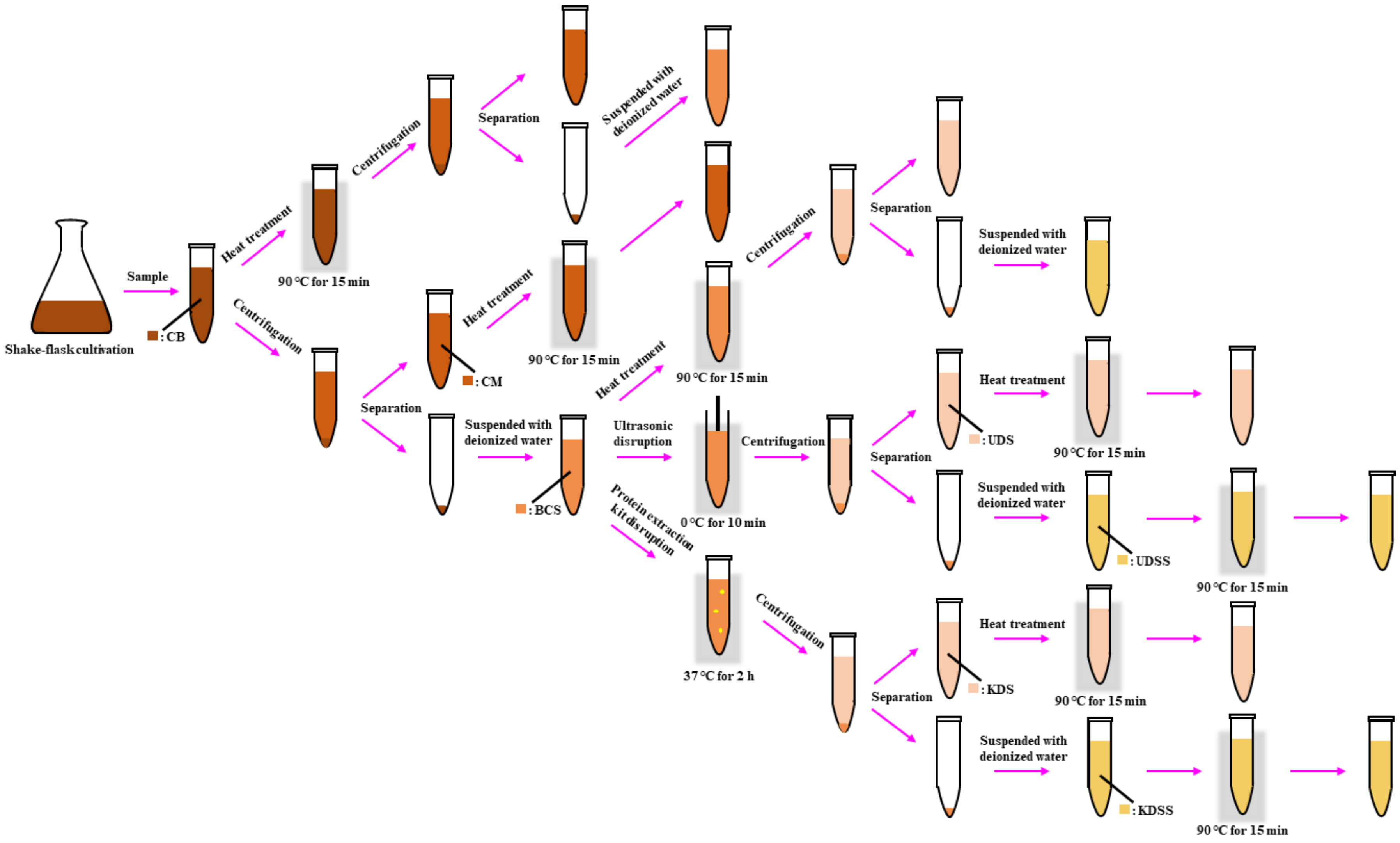
Shake-flask cultivation
Sample
: CB
Heat treatment
90 °C for 15 min
Centrifugation
Separation
Suspended with deionized water
: CM
Heat treatment
90 °C for 15 min
: BCS
Ultrasonic disruption
Heat treatment
90 °C for 15 min
0 °C for 10 min
Protein extraction kit disruption
37 °C for 2 h
Centrifugation
Separation
Suspended with deionized water
Heat treatment
: UDS
90 °C for 15 min
: UDSS
: KDS
: KDSS

7

Fig. S4 Peptides obtained from a MALDI-TOF/TOF analysis of the high-molecular-weight inclusion bodies from *P. furiosus* α-amylase production in *B. subtilis*. The amino acid sequence of *P. furiosus* α-amylase is provided below; matching peptides are underlined. Sequence coverage was 24%

```
1    AKYLELEEGGVIMQAFYWDVPGGGIWWDHIRSKIPEWYEAGISAIWLPPP
51   SKGMSGGYSMGYDPYDYFDLGEYYQKGTVETRFGSKEELVRLIQTAHAYG
101  IKVIADVVINHRAGGDLEWNPFVGDYTWTDFSKVASGKYTANYLDFHPNE
151  LHCCDEGTFGGFPDICHHKEWDQYWLWKSNESYAAYLRSIGFDGWRFDYV
201  KGYGAWVVRDWLNWWGGWAVGEYWDTNVDALLSWAYESGAKVFDFPLYYK
251  MDEAFDNNNIPALVYALQNGQTVVSRDPFKAVTFVANHDTDIIWNKYPAY
301  AFILTYEGQPVIFYRDFEEWLNKDKLINLIWIHDHLAGGSTTIVYYDNDE
351  LIFVRNGDSRRPGLITYINLSPNWVGRWVYVPKFAGACIHEYTGNLGGWV
401  DKRVDSSGWVYLEAPPHDPANGYYGYSVWSYCGVG
```

Fig. S5 Peptides obtained from a MALDI-TOF/TOF analysis of the low-molecular-weight inclusion bodies from *P. furiosus* α-amylase production in *B. subtilis*. The amino acid sequence of *P. furiosus* α-amylase is provided below; matching peptides are underlined. Sequence coverage was 29%

```
1    AKYLELEEGGVIMQAFYWDVPGGGIWWDHIRSKIPEWYEAGISAIWLPPP
51   SKGMSGGYSMGYDPYDYFDLGEYYQKGTVETRFGSKEELVRLIQTAHAYG
101  IKVIADVVINHRAGGDLEWNPFVGDYTWTDFSKVASGKYTANYLDFHPNE
151  LHCCDEGTFGGFPDICHHKEWDQYWLWKSNESYAAYLRSIGFDGWRFDYV
201  KGYGAWVVRDWLNWWGGWAVGEYWDTNVDALLSWAYESGAKVFDFPLYYK
251  MDEAFDNNNIPALVYALQNGQTVVSRDPFKAVTFVANHDTDIIWNKYPAY
301  AFILTYEGQPVIFYRDFEEWLNKDKLINLIWIHDHLAGGSTTIVYYDNDE
351  LIFVRNGDSRRPGLITYINLSPNWVGRWVYVPKFAGACIHEYTGNLGGWV
401  DKRVDSSGWVYLEAPPHDPANGYYGYSVWSYCGVG
```

References

1. Zhang K, Su L, Duan X, Liu L, Wu J. 2017. High-level extracellular protein production in *Bacillus subtilis* using an optimized dual-promoter expression system. *Microb Cell Fact.* **16:** 32.
